# Functional Coupling between the Unfolded Protein Response and Endoplasmic Reticulum/Golgi Ca^2+^-ATPases Promotes Stress Tolerance, Cell Wall Biosynthesis, and Virulence of Aspergillus fumigatus

**DOI:** 10.1128/mBio.01060-20

**Published:** 2020-06-02

**Authors:** Martin Weichert, José Guirao-Abad, Vishukumar Aimanianda, Karthik Krishnan, Christina Grisham, Patrick Snyder, Alex Sheehan, Ruthvik R. Abbu, Hong Liu, Scott G. Filler, Eric I. Gruenstein, Jean-Paul Latgé, David S. Askew

**Affiliations:** a Department of Pathology & Laboratory Medicine, University of Cincinnati College of Medicine, Cincinnati, Ohio, USA; b Institut Pasteur, Molecular Mycology Unit, CNRS, UMR2000, Paris, France; c Division of Infectious Diseases, Lundquist Institute for Biomedical Innovation at Harbor-UCLA Medical Center, Torrance, California, USA; d Department of Molecular Genetics, Biochemistry & Microbiology, University of Cincinnati College of Medicine, Cincinnati, Ohio, USA; e Unité des Aspergillus, Institut Pasteur, Paris, France; Duke University Medical Center

**Keywords:** *Aspergillus fumigatus*, UPR, HacA, ER stress, calcium, SERCA, SPCA, cell wall, galactomannan

## Abstract

Many species of pathogenic fungi deploy the unfolded protein response (UPR) to expand the folding capacity of the endoplasmic reticulum (ER) in proportion to the demand for virulence-related proteins that traffic through the secretory pathway. Although Ca^2+^ plays a pivotal role in ER function, the mechanism by which transcriptional upregulation of the protein folding machinery is coordinated with Ca^2+^ homeostasis is incompletely understood. In this study, we investigated the link between the UPR and genes encoding P-type Ca^2+^-ATPases in the human-pathogenic mold Aspergillus fumigatus. We demonstrate that acute ER stress increases transcription of the *srcA* gene, encoding a member of the sarco/endoplasmic reticulum Ca^2+^-ATPase (SERCA) family, as well as that of *pmrA*, encoding a secretory pathway Ca^2+^-ATPase (SPCA) in the Golgi membrane. Loss of the UPR transcription factor HacA prevented the induction of *srcA* and *pmrA* transcription during ER stress, defining these ER/Golgi Ca^2+^ pumps as novel downstream targets of this pathway. While deletion of *srcA* alone caused no major deficiencies, a Δ*srcA*/Δ*pmrA* mutant displayed a severe polarity defect, was hypersensitive to ER stress, and showed attenuated virulence. In addition, cell wall analyses revealed a striking reduction in mannose levels in the absence of both Ca^2+^ pumps. The Δ*hacA* mutant was hypersensitive to agents that block calcineurin-dependent signaling, consistent with a functional coupling between the UPR and Ca^2+^ homeostasis. Together, these findings demonstrate that the UPR integrates the need for increased levels of chaperone and folding enzymes with an influx of Ca^2+^ into the secretory pathway to support fungal growth, stress adaptation, and pathogenicity.

## Abstract (precis)

The UPR is an intracellular signal transduction pathway that maintains homeostasis of the ER. The pathway is also tightly linked to the expression of virulence-related traits in diverse species of human-pathogenic and plant-pathogenic fungal species, including the predominant mold pathogen infecting humans, Aspergillus fumigatus. Despite advances in the understanding of UPR signaling, the linkages and networks that are governed by this pathway are not well defined. In this study, we revealed that the UPR is a major driving force for stimulating Ca^2+^ influx at the ER and Golgi membranes and that the coupling between the UPR and Ca^2+^ import is important for virulence, cell wall biosynthesis, and resistance to antifungal compounds that inhibit Ca^2+^ signaling.

## INTRODUCTION

Filamentous fungi contribute to the beneficial decomposition of organic matter in the environment but may also be detrimental to plants, animals, and humans through infection (1). These fungal lifestyles are largely supported by a highly developed endoplasmic reticulum (ER) that enables the secretion of degradative enzymes and effector proteins, which can damage host tissues during infection (2–5). Many of these environmental molds propagate themselves by the release of conidia (spores) into the atmosphere, making exposure an inevitable consequence of daily life (1, 6). Aspergillus fumigatus is an important component of this airborne biomass because it represents the predominant mold pathogen infecting humans (7). In healthy individuals, inhaled A. fumigatus conidia are cleared from the lung by innate pulmonary defenses. However, in patients with compromised immune systems or individuals with preexisting structural lung disease, delayed clearance allows the conidia to germinate into hyphae. Secretion of digestive enzymes by germinating spores and hyphae promotes invasion of the lung tissue, resulting in a life-threatening infection known as invasive aspergillosis (IA). The outcome of IA is very poor, even when treated, with mortality rates exceeding 50% (8).

All eukaryotic cells that are specialized for secretion possess an abundant ER and Golgi apparatus that are collectively responsible for the proper folding, assembly, modification, and delivery of proteins into the extracellular milieu. Protein folding is accomplished by molecular chaperones that transiently interact with nascent polypeptides as they enter the ER, as well as folding or processing enzymes in the ER/Golgi compartments that stabilize protein conformations through posttranslational modifications such as glycosylation and disulfide bridge formation (9, 10). However, when the demand for secretion exceeds the protein folding capacity of the ER, the ensuing accumulation of unfolded or misfolded proteins can generate proteotoxic stress. Fungi, like other eukaryotic cells, rely on a stress response pathway known as the unfolded protein response (UPR) to provide adaptive outputs that adjust the functionality of the ER folding machinery in response to fluctuating demands (5, 11, 12). Interestingly, both plant-pathogenic and human-pathogenic fungi also exploit the UPR as a regulatory hub to control the expression of virulence-related traits, such as thermotolerance, iron acquisition, cell wall homeostasis, hypoxia adaptation, effector secretion, biofilm formation, resistance to antimicrobial peptides, and antifungal drug susceptibility (13–24). The A. fumigatus UPR follows the basic paradigm of fungal UPR signaling established in pioneering studies in the model yeast Saccharomyces cerevisiae (25). The proximal stress sensor of the pathway is a type I ER transmembrane protein known as IreA in A. fumigatus. Current evidence indicates that the sensor becomes activated by interaction with the unfolded proteins that accumulate in the ER lumen during ER stress (26). These interactions trigger oligomerization in the membrane and activation of a cytoplasmic endoribonuclease (RNase) domain. The RNase then cleaves an unconventional intron from a cytosolic mRNA, creating a frameshift that specifies the translation of a bZIP transcription factor known as HacA in A. fumigatus (25). HacA coordinates a network of transcriptional changes to enhance the folding capacity of the ER, including the upregulation of mRNAs encoding ER-resident chaperones and protein folding or processing enzymes, many of which require calcium ions (Ca^2+^) as an essential cofactor (13, 27).

The concentration of Ca^2+^ in the ER lumen is maintained at several orders of magnitude higher than in the cytoplasm. In mammalian cells, this gradient differential is largely accomplished by the action of two major families of membrane P-type Ca^2+^-ATPases: the sarco/endoplasmic reticulum Ca^2+^-ATPases (SERCA) located in the ER/early Golgi compartments and the secretory pathway Ca^2+^/Mn^2+^-ATPases (SPCA) located in the trans-Golgi network (28). The mammalian SERCA pump is encoded by three genes, each of which generates additional isoforms with distinct enzymatic properties and expression profiles, making this one of the most diverse and important families of P-type ATPases, with notable functions in muscle physiology that are relevant to human disease (28). In contrast to S. cerevisiae, where no SERCA pumps are found, the genome of A. fumigatus is predicted to encode a single uncharacterized SERCA homolog, designated SrcA here (29). In this study, we examined the contribution of SrcA to stress adaptation and virulence of A. fumigatus. The results demonstrate that the UPR coordinates increased transcription of the *srcA* gene, as well as of the gene encoding the SPCA homolog PmrA, in a HacA-dependent manner and that expression of these Ca^2+^ pumps jointly contributes to the ability of A. fumigatus to adapt to ER stress, to maintain cell wall integrity, and to cause infection.

## RESULTS

### The UPR directs increased expression of genes encoding SERCA and SPCA Ca^2+^ pumps during ER stress.

**FIG 1:**
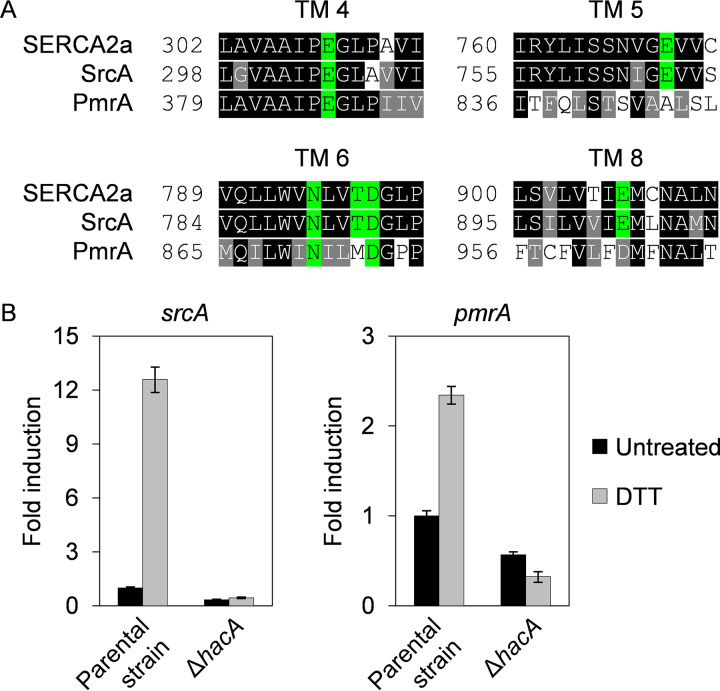
The UPR is required for transcriptional upregulation of genes encoding P-type Ca^2+^-ATPases during ER stress. (A) Amino acid sequence alignment of Ca^2+^-binding sites within transmembrane (TM) domains of the human ER Ca^2+^-ATPase SERCA2a (ATP2A2A) with the A. fumigatus SERCA homolog SrcA (Afu6g06740) and the A. fumigatus SPCA homolog PmrA (Afu2g05860). Residues indicated in green are completely conserved in SERCA-type Ca^2+^-ATPases. (B) RT-qPCR analysis of *srcA* and *pmrA* expression in cultures of the Δ*hacA* mutant and its KU70 parental strain grown in liquid YG medium for 16 h at 37°C and 200 rpm. ER stress was induced by treatment with 1 mM DTT for 1 h prior to harvest. Values represent means ± SD of results from three technical replicates from one representative experiment performed as described in Materials and Methods.

The A. fumigatus gene *srcA* (Afu6g06740) codes for a protein that is most closely related to the SERCA2a isoform of human SERCA homologs. The A. fumigatus SrcA protein displays similar overall characteristics of predicted domain organization, topology, and conservation of residues in transmembrane helices 4, 5, 6, and 8, which are pivotal to Ca^2+^ transfer across the ER membrane (Fig. 1A; see also Fig. S1A and B in the supplemental material). *In situ* tagging of the *srcA* gene with *egfp* revealed a predominant perinuclear ER localization (Fig. S1C), in accordance with the expected ER membrane localization of SERCA homologs in fungal species (30, 31). Since Ca^2+^ import into the ER is necessary for an optimal folding environment (32), we hypothesized that the *srcA* gene would be under the control of the UPR. To test this, quantitative reverse transcription PCR (RT-qPCR) analyses were performed after treatment with dithiothreitol (DTT), a compound that causes acute ER stress by reducing the disulfide bonds that stabilize many secreted proteins (33). Transcription of *srcA* increased more than 10-fold, which was associated with an increase in SrcA protein (Fig. 1B; see also Fig. S1C and D), suggesting that an increase in SrcA levels is needed to supply additional Ca^2+^ during ER stress. Importantly, no induction was observed in a Δ*hacA* mutant that lacked the transcription factor necessary for UPR activation (Fig. 1B), demonstrating that *srcA* is a newly identified transcriptional target of the UPR in A. fumigatus.

10.1128/mBio.01060-20.1 FIG S1 Domain organization, topology, and localization of SrcA in A. fumigatus. (A) Full amino acid alignment comparing the human ER Ca^2+^-ATPase SERCA2a (ATP2A2A; UniProtKB identifier P16615-2) with the SERCA homolog SrcA in A. fumigatus (Afu6g06740; retrieved from reference strain Af293 at FungiDB), illustrating conservation of the A (actuator), N (nucleotide binding), and P (phosphorylation) domains as well as transmembrane (TM) helices established in SERCA2a. Asterisks (*) indicate residues crucial for ATP binding, hash marks (#) show the conserved phosphorylation motif, and plus signs (+) mark a motif essential for dephosphorylation. Amino acids indicated in green are pivotal to Ca^2+^ transfer across the ER membrane. The alignment was generated with the Web tools T-coffee (http://tcoffee.crg.cat/apps/tcoffee/do:regular) and Boxshade (http://www.ch.embnet.org/software/BOX_form.html). The positions of transmembrane helices, functional domains, and residues involved in Ca^2+^ transport were derived from the literature. (B) *In silico* prediction of the topologies of human SERCA2a and A. fumigatus SrcA using the Web tool TOPCONS (http://topcons.cbr.su.se/pred/). (C) *In situ* tagging of SrcA with enhanced green fluorescent protein (eGFP) reveals typical perinuclear ER localization. Bright-field microscopy (top) and fluorescence microscopy (bottom) images show hyphae grown in liquid AMM after 18 h at 30°C. To induce ER stress, samples were incubated for 1 h with 1 mM DTT prior to imaging. Bars: 10 μm. (D) Immunoblot analysis of SrcA-eGFP. Total protein was extracted from mycelium grown in liquid YG medium for 16 h at 37°C and 200 rpm. ER stress was induced with 1 mM DTT for 1 h prior to harvest. The nitrocellulose membrane was first hybridized with the D5.1 anti-GFP primary antibody and an anti-rabbit horseradish peroxidase (HRP)-conjugated secondary antibody to detect chemiluminescence signals of SrcA-eGFP (expected size: 136 kDa), followed by reprobing with the DM1A anti-α-tubulin primary antibody and an anti-mouse HRP-linked secondary antibody (Cell Signaling Technology). M: photos of a prestained marker for protein sizes. Download FIG S1, JPG file, 2.3 MB. Copyright © 2020 Weichert et al. 2020 Weichert et al. This content is distributed under the terms of the Creative Commons Attribution 4.0 International license.

Since protein processing enzymes in the Golgi compartment also require Ca^2+^ for optimal function (28), we hypothesized that UPR control of Ca^2+^ import would extend beyond the ER into the Golgi compartment. The yeast Golgi Ca^2+^ pump Pmr1 was the first SPCA protein to be identified, and its ortholog in A. fumigatus is PmrA (28, 34). As shown in Fig. 1B, expression of the *pmrA* gene was also induced by DTT treatment, albeit to a lesser extent than *srcA*. As observed for *srcA*, the upregulation of *pmrA* seen under conditions of ER stress was also blocked in the Δ*hacA* mutant (Fig. 1B), indicating that both of these Ca^2+^ pump genes are under the transcriptional control of the canonical UPR pathway. The induction of *srcA* and *pmrA* under conditions of ER stress was confirmed in a distinct A. fumigatus isolate (Fig. S2A). However, no UPR-dependent induction was observed for *pmcA*, *pmcB*, and *pmcC* (Fig. S3A), representing genes encoding members of the plasma membrane Ca^2+^-ATPase (PMCA) family, which localize to cell or vacuolar membranes in fungi (29, 31, 35). These results demonstrate that ER stress triggers the UPR-dependent transcriptional induction of *srcA* and *pmrA* genes, providing a mechanism to increase ER/Golgi Ca^2+^ levels in parallel with the UPR-directed rise in expression of Ca^2+^-dependent chaperones and folding enzymes.

10.1128/mBio.01060-20.2 FIG S2 Loss of *srcA* or *pmrA* does not induce compensatory reciprocal gene expression or induction of genes encoding P-type Ca^2+^-ATPases. (A) RT-qPCR analysis shows that KU80, the second parental strain used in this study, exhibited the same induction of the *srcA* and *pmrA* genes as KU70 (see Fig. 1). Cultures were grown in liquid YG medium for 16 h at 37°C followed by treatment with 1 mM DTT for 1 h prior to harvest. Bars show means ± SD of results from four (*srcA*) and three (*pmrA*) biological replicates per condition (**, *P < *0.01; ****, *P < *0.0001 [unpaired, two-tailed *t* tests]). (B) RT-qPCR analysis shows that loss of either *pmrA* or *srcA* does not trigger compensatory upregulation of the reciprocal gene. The strains were grown as described in the panel A legend but were treated with DTT for only 30 min. (C) RT-qPCR analysis showing that loss of *srcA* and *pmrA* is not associated with compensatory induction of genes encoding three members of the PMCA family of Ca^2+^-ATPases in the presence or absence of ER stress. The mycelia were grown for 12 h (KU80 parental strain) or 24 h (Δ*srcA*/Δ*pmrA* mutant) at 37°C in liquid YG medium, followed by treatment with 1 mM DTT prior to harvest. Values in panels B and C represent means ± SD of results from three technical replicates from one representative experiment performed as described in Materials and Methods. Download FIG S2, JPG file, 0.7 MB. Copyright © 2020 Weichert et al. 2020 Weichert et al. This content is distributed under the terms of the Creative Commons Attribution 4.0 International license.

10.1128/mBio.01060-20.3 FIG S3 The expression profiles of *srcA* and *pmrA* differ from those of the *pmcA-C* genes. (A) Genes *pmcA*, *pmcB*, and *pmcC* do not show HacA-dependent induction profiles during ER stress. ER stress was induced by treating overnight cultures (16 h) of the indicated strains in YG medium at 37°C with 1 mM DTT for 1 h prior to harvest for RT-qPCR analysis. Parental strain: KU70. (B) The *srcA* and *pmrA* genes do not show the Ca^2+^-dependent induction characteristic of *pmc* genes. Parental strain KU70 was grown in liquid AMM for 20 h at 37°C prior to the addition of 50 mM CaCl_2_. RNA was extracted after 5 and 15 min for RT-qPCR analysis. Values represent means ± SD of results from three technical replicates from one representative experiment performed as described in Materials and Methods. Download FIG S3, JPG file, 0.4 MB. Copyright © 2020 Weichert et al. 2020 Weichert et al. This content is distributed under the terms of the Creative Commons Attribution 4.0 International license.

### SrcA and PmrA promote radial growth and conidiation.

**FIG 2:**
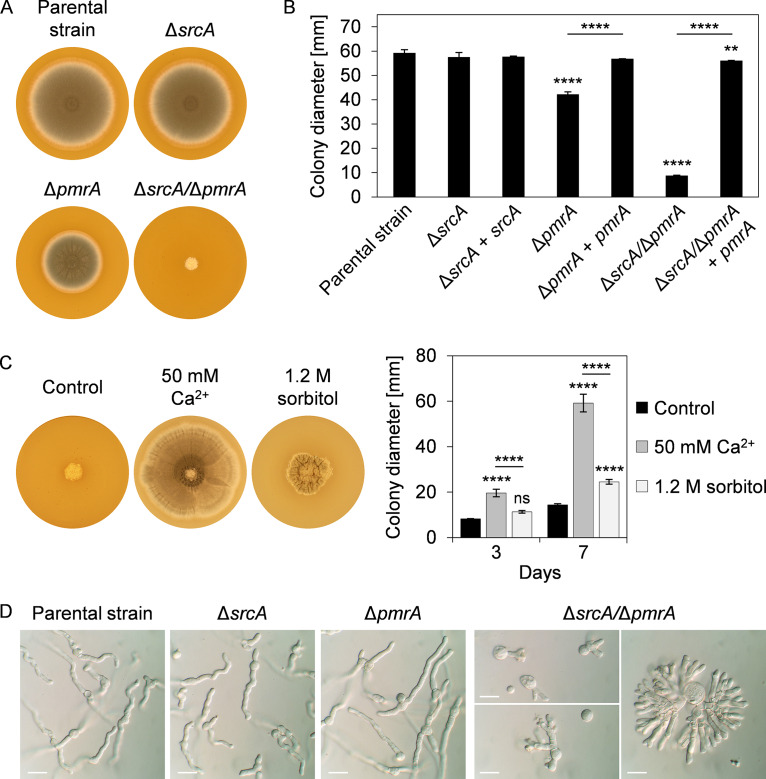
SrcA and PmrA jointly support polarized growth of A. fumigatus. (A) Colony morphology of the indicated strains after 3 days of growth at 37°C on IMA plates. (B) Colony diameters after 3 days of growth on IMA plates at 37°C. (C) Colony morphology of the Δ*srcA*/Δ*pmrA* mutant after 7 days of culture at 37°C on IMA plates in the presence or absence of added Ca^2+^ or sorbitol. Quantitation of colony diameters after 3 and 7 days is shown to the right. (D) Microscopic analysis of the morphology of hyphae grown for 10 h (KU80 parental strain, Δ*srcA* mutant, Δ*pmrA* mutant) or 13 to 20 h (Δ*srcA*/Δ*pmrA* double mutant) at 37°C in liquid YG medium. Bars: 20 μm. Values in panels B and C represent means ± SD (**, *P < *0.01; ****, *P < *0.0001; ns, not significant [one-way ANOVA with Tukey’s *post hoc* test]).

We next deleted the A. fumigatus *srcA* and *pmrA* genes individually and in combination (Fig. S4A; see also Fig. S5A). The Δ*srcA* mutant was phenotypically indistinguishable from the parental strain at 37°C on either complex or minimal medium (Fig. 2A and B; see also Fig. S6A). The Δ*pmrA* mutant was slightly growth impaired, confirming what was previously reported for this mutant (34). In contrast, a Δ*srcA*/Δ*pmrA* double deletion mutant revealed a severe growth defect, producing tightly restricted colonies that lacked conidia and were barely able to expand radially. Complementation of these mutants restored growth to normal levels (Fig. 2B). Moreover, supplementation of the medium with Ca^2+^ allowed the Δ*srcA*/Δ*pmrA* mutant to completely fill the plate and produce conidia (Fig. 2C). However, osmotic stabilization of the medium with sorbitol did not rescue radial growth to the same extent as addition of Ca^2+^, suggesting that the abnormal colony morphology of the Δ*srcA*/Δ*pmrA* mutant was primarily a consequence of altered Ca^2+^ homeostasis rather than of osmotic imbalance.

10.1128/mBio.01060-20.4 FIG S4 Validation of the Δ*srcA* and Δ*pmrA* single mutants of A. fumigatus. (A) (Left panels) PCR confirmation of *srcA* gene modification using primer pairs specific for the wild-type locus (1007 + 1008) and the deletion (1073 + 1012). Primer locations are shown in the schematic to the right. Left-arm (LA) and right-arm (RA) regions undergo homologous recombination with the knockout construct p670, leaving behind a *six* site after β-recombinase-directed excision of the marker module from the locus as detailed in Materials and Methods. The complementation of the Δ*srcA* mutant was accomplished by reintegration of the gene at its native locus. (B) PCR confirmation of *pmrA* gene modification using primers that are specific for the wild-type locus (1164 + 1165) or that distinguish between native and deleted sequences (1166 + 1258). Complementation was accomplished by integrating the *pmrA* gene in a site-specific manner into an intergenic region (IR) revealed with primers 1082 and 1215. Primer locations are shown in the schematic to the right. (C) Complementation of the Δ*srcA* and Δ*pmrA* double mutant rescues growth under thermal stress conditions. Conidia were spotted onto the center of AMM plates and incubated for 7 days at 37°C and 45°C. The relative percentages of growth (mean values ± SD) were calculated from three plates per strain and condition (***, *P < *0.001; ****, *P < *0.0001 [one-way ANOVA with Tukey’s *post hoc* test]). (D) Complementation rescues sensitivity to cell wall perturbation. Serial 10-fold dilutions of conidia from the indicated strains were spotted onto AMM plates in the presence of calcofluor white (CFW), Congo red (CR), or hygromycin B (HygB) and incubated for 2 days at 37°C. Download FIG S4, JPG file, 1.6 MB. Copyright © 2020 Weichert et al. 2020 Weichert et al. This content is distributed under the terms of the Creative Commons Attribution 4.0 International license.

10.1128/mBio.01060-20.5 FIG S5 Validation of the Δ*srcA*/Δ*pmrA* double mutant of A. fumigatus. (A) PCR confirmation of the expected genotype of the Δ*srcA*/Δ*pmrA* double mutant with primer pairs specific for the knockout of *srcA* and *pmrA*, respectively. Primer numbers and locations are shown at the bottom and in Fig. S4A. The *pmrA* gene in the Δ*srcA* mutant was replaced by homologous recombination performed with the knockout construct p691 composed of left arms (LA) and right arms (RA) flanking the native locus and the *six*-site/β-recombinase/chlorimuron-ethyl resistance (*cme*^R^) marker module as described in Materials and Methods. Reintegration of the *pmrA* gene into the double mutant was accomplished by recombining vector p701 with an intergenic region (see Fig. S4B). (B) Complementation of the Δ*srcA*/Δ*pmrA* double mutant with the *pmrA* gene rescues stress hypersensitivity. Serial 10-fold dilutions of conidia from the indicated strains were spotted onto AMM plates containing various stress agents and incubated for 2 days. Download FIG S5, JPG file, 1.6 MB. Copyright © 2020 Weichert et al. 2020 Weichert et al. This content is distributed under the terms of the Creative Commons Attribution 4.0 International license.

10.1128/mBio.01060-20.6 FIG S6 Loss of SrcA and PmrA increases sensitivity to low external Ca^2+^. (A) The severe growth defect and hyperbranching phenotypes of the Δ*srcA*/Δ*pmrA* mutant on rich medium (IMA or YG; see Fig. 2) are recapitulated on minimal medium (AMM). AMM plates (top) were incubated for 7 days at 37°C. Cultures were incubated in liquid AMM (bottom) for 12 h (the KU80 parental strain and the Δ*srcA* and Δ*pmrA* mutant strains) or 28 h (the Δ*srcA*/Δ*pmrA* double mutant) at 37°C. Bars: 20 μm. (B) Hyperbranching of the parental strain in liquid AMM supplemented with 300 μM BAPTA after 22 h at 37°C. Bar: 20 μm. (C) The Δ*pmrA* mutant, but not the Δ*srcA* mutant, is hypersensitive to Ca^2+^ chelation by BAPTA (100 μM). Conidia were inoculated into the center of AMM plates in the presence or absence of BAPTA and incubated for 7 days at 37°C. For each strain, the percentages of colony diameters of treated versus untreated mycelia (mean values ± SD) were calculated from triplicate plates (****, *P < *0.0001 [one-way ANOVA with Tukey’s *post hoc* test]). (D) The Δ*srcA*/Δ*pmrA* strain is more sensitive to low Ca^2+^ than the other strains, and complementation of *pmrA* in the Δ*srcA*/Δ*pmrA* strain reverses this sensitivity. Serial 10-fold dilutions of conidia from the indicated strains were spotted onto AMM plates in the presence or absence of BAPTA and incubated for 2 days at 37°C. Download FIG S6, JPG file, 1.4 MB. Copyright © 2020 Weichert et al. 2020 Weichert et al. This content is distributed under the terms of the Creative Commons Attribution 4.0 International license.

No differences in hyphal morphology were evident in liquid cultures of the two single gene deletion strains in either rich medium (Fig. 2D) or minimal medium (Fig. S6A). However, the Δ*srcA*/Δ*pmrA* mutant was defective in polarized growth, resulting in exaggerated isotropic swelling of germinating conidia and in hyperbranching of germ tubes (Fig. 2D; see also Fig. S6A). Interestingly, a similar hyperbranching phenotype could be induced in the parental strain by reducing Ca^2+^ levels with the cell-impermeant Ca^2+^-selective chelator BAPTA (Fig. S6B). In contrast to the Δ*srcA* mutant, both the Δ*pmrA* and the Δ*srcA*/Δ*pmrA* mutants were hypersensitive to BAPTA, suggesting a more prominent role for PmrA under conditions of Ca^2+^ limitation (Fig. S6C and D). We conclude that SrcA and PmrA share overlapping roles in Ca^2+^ homeostasis in the secretory pathway and that these functions support both conidiation and polarized growth in A. fumigatus.

### Loss of SrcA and PmrA exacerbates ER stress.

**FIG 3:**
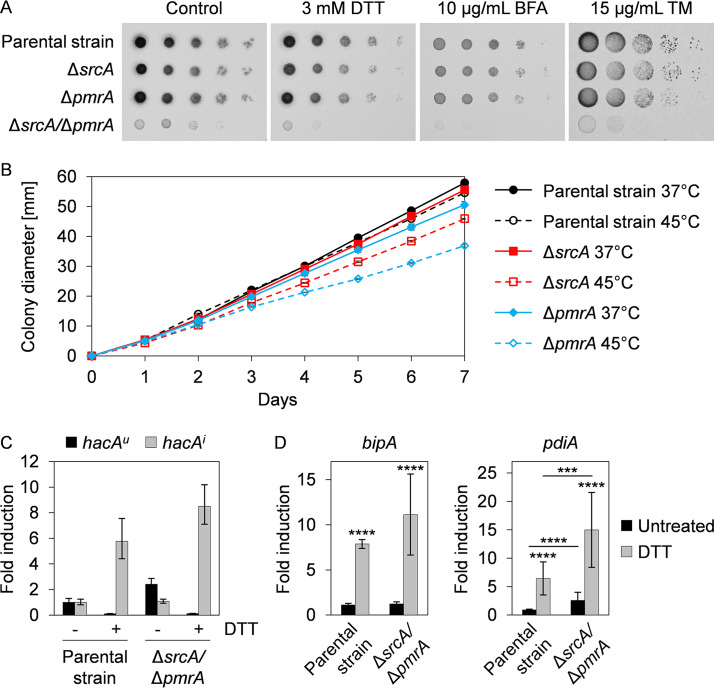
The Δ*srcA*/Δ*pmrA* mutant is hypersensitive to ER stress. (A) Serial dilutions of conidia (10^5^ to 10) from the indicated strains were incubated for 2 days at 37°C on AMM plates supplemented with DTT, BFA, or TM. (B) Conidia were spotted onto the center of AMM plates and incubated for 7 days at 37°C or 45°C. Curves represent mean values ± SD of results from three plates per strain and condition. (C) The canonical UPR is functional in the Δ*srcA*/Δ*pmrA* mutant. Graphs show the fold change in the level of *hacA^u^* and *hacA^i^* mRNAs by RT-qPCR after treatment of overnight cultures in liquid YG medium at 37°C (12 h for the KU80 parental strain and 24 h for the Δ*srcA*/Δ*pmrA* mutant) with 1 mM DTT for 15 min. Values represent means ± SD of results from three technical replicates from one representative experiment. (D) Fold change in the levels of UPR target genes *bipA* and *pdiA* after 60 min of treatment with 1 mM DTT. Bars show means ± SD of results from five biological replicates per strain and condition (***, *P < *0.001; ****, *P < *0.0001 [one-way ANOVA with Tukey’s *post hoc* test]).

Since SERCA proteins have important roles in maintaining ER homeostasis in mammals and fungi (28, 36), we were surprised to find that the Δ*srcA* and Δ*pmrA* mutants showed no increase in sensitivity to chemical inducers of the UPR, including DTT, tunicamycin (TM), and brefeldin A (BFA) (Fig. 3A). However, both strains exhibited reduced growth in the presence of thermal stress (Fig. 3B; see also Fig. S4C), which is a condition that is known to perturb protein folding efficiency and involve UPR intervention (25). In contrast, the Δ*srcA*/Δ*pmrA* mutant was hypersensitive to both chemically and thermally induced ER stress (Fig. 3A; see also Fig. S5B). The enhanced susceptibility of the double mutant to ER stress was not due to a failure to activate the canonical UPR, as shown by the ability of DTT to trigger induction of the *hacA^i^* mRNA (Fig. 3C). However, the double mutant revealed a higher level of expression of at least one known UPR target gene, that encoding the protein disulfide isomerase PdiA, suggesting that ER stress levels are exacerbated in this strain (Fig. 3D). We conclude that SrcA and PmrA are functionally redundant under conditions of ER stress but that a decline in Ca^2+^ availability caused by their combined absence impairs the folding capacity of the ER and intensifies the level of ER stress in the fungus.

### Cell wall composition and homeostasis are supported by SrcA and PmrA.

**FIG 4:**
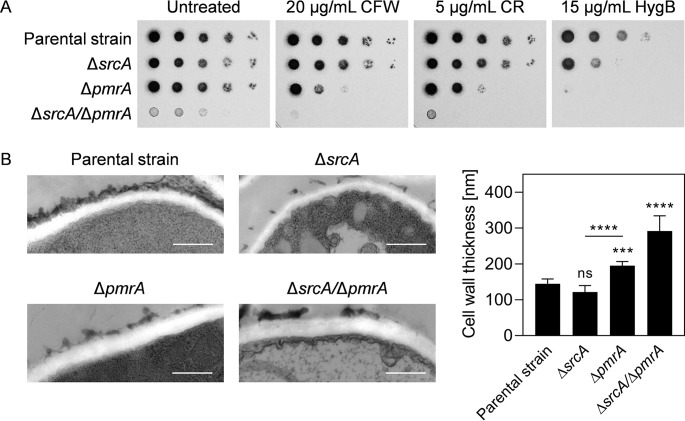
Loss of SrcA and PmrA disrupts cell wall integrity and structure. (A) Serial 10-fold dilutions of conidia from the indicated strains were spotted onto AMM plates containing calcofluor white (CFW), Congo red (CR), or hygromycin B (HygB) and incubated for 2 days at 37°C. (B) TEM analysis of cross sections of hyphae grown for 16 h at 37°C in liquid YG medium. Representative TEM images (left panels) and quantitative analysis of cell wall thickness (right) are presented. Values represent means ± SD (***, *P < *0.001; ****, *P < *0.0001; ns, not significant [one-way ANOVA with Tukey’s *post hoc* test]). Bars: 500 nm (magnification of ×50,000).

Unlike the Δ*srcA* mutant, both the Δ*pmrA* and Δ*srcA*/Δ*pmrA* mutants were hypersensitive to cell wall perturbation with calcofluor white (CFW) and Congo red (CR) (Fig. 4A). This could be rescued by reconstitution of *pmrA* (Fig. S4D; see also Fig. S5B), demonstrating that PmrA has the dominant effect on cell wall integrity between these two Ca^2+^ pumps. However, morphological analysis of the hyphal cell wall by transmission electron microscopy (TEM) revealed a greater thickening of the cell wall in the Δ*srcA*/Δ*pmrA* mutant than in the Δ*pmrA* strain, signifying a cooperating role for SrcA in the maintenance of cell wall structure (Fig. 4B). Since glycosylation events are central to cell wall biosynthesis, we compared the sensitivities of the mutants to hygromycin B, a compound that has increased toxicity for glycosylation-defective mutants (37, 38). Both the Δ*srcA* mutant and the Δ*pmrA* mutant were hypersensitive to this compound, consistent with the notion that the loss of either of these genes creates a defect in glycosylation events in the ER/Golgi compartments.

**FIG 5:**
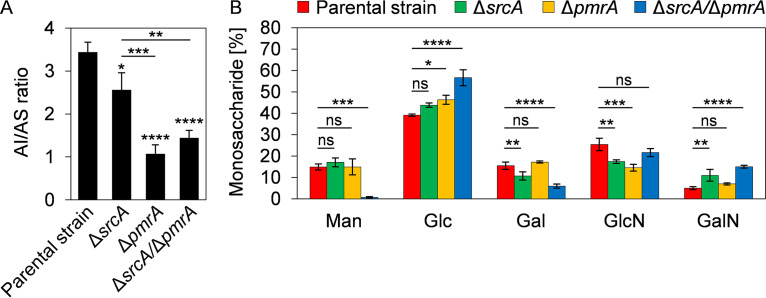
Loss of SrcA and PmrA alters the monosaccharide composition of the cell wall. (A) Ratios of alkali-insoluble (AI) and alkali-soluble (AS) fractions of the hyphal cell wall in the indicated strains obtained from mycelia that were grown in liquid YPD medium for 40 h at 37°C. (B) Quantitative analysis of the total monosaccharide composition in the mycelial cell wall of the indicated strains. Values in panels A and B represent means ± SD of results from three independent cultures per strain (*, *P < *0.05; **, *P < *0.01; ***, *P < *0.001; ****, *P* < 0.0001; ns, not significant [one-way ANOVA with Tukey’s *post hoc* test in panel A and Dunnett’s *post hoc* test in panel B]). Man, mannose; Glc, glucose; Gal, galactose; GlcN, glucosamine; GalN, galactosamine.

Biochemically, the hyphal cell wall of A. fumigatus can be divided into two fractions: an alkali-soluble (AS) fraction comprised primarily of α(1,3)-glucan and galactosaminogalactan and an alkali-insoluble (AI) fraction comprised of β(1,3)-glucan and chitin, with galactomannan being present in both fractions (39). A decrease in the AI/AS ratio was observed in the Δ*srcA* mutant, but the effect was more pronounced in the Δ*pmrA* and Δ*srcA*/Δ*pmrA* mutants (Fig. 5A). Analysis of the monosaccharide composition of the cell wall revealed abnormalities in the Δ*srcA* mutant and the Δ*pmrA* mutant relative to the parental strain (Fig. 5B). However, the combined losses of SrcA and PmrA had the strongest impact on cell wall composition, resulting in the near-absence of mannose in the cell wall (Fig. 5B), suggesting a loss of galactomannan. We conclude that alterations in Ca^2+^ homeostasis in the ER/Golgi compartments caused by the absence of these UPR-dependent Ca^2+^ pumps impair the synthesis and/or delivery of crucial cell wall components, resulting in abnormal structure and biochemical composition, particularly with respect to mannose.

### SrcA and PmrA provide support for the virulence of A. fumigatus.

**FIG 6:**
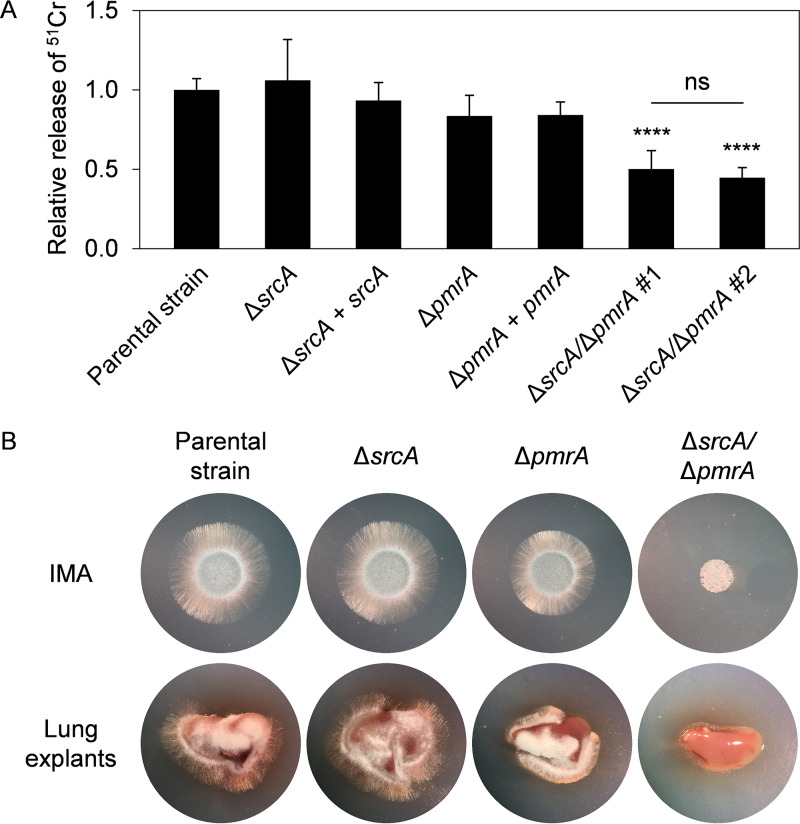
SrcA and PmrA jointly contribute to epithelial cell damage and growth on lung tissue. (A) Relative levels of release of ^51^Cr from A549 epithelial cells challenged for 24 h with the indicated strains. Two isolates of the double mutant were tested side by side. Mean values ± SD were calculated from four independent experiments (****, *P < *0.0001; ns, not significant [one-way ANOVA with Tukey’s *post hoc* test]). (B) Lung explants of untreated female CF-1 mice were placed onto plates lacking nutrients (water with agarose) and inoculated with 2 × 10^3^ conidia of the indicated strains, and IMA plates were run in parallel as growth controls. All plates were incubated for 30 h at 37°C.

**FIG 7:**
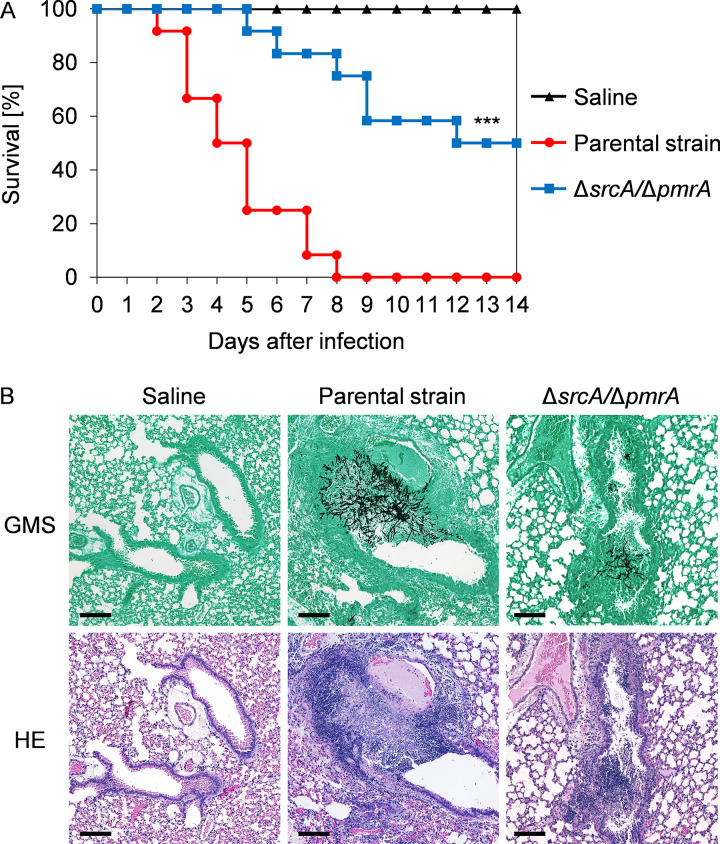
SrcA and PmrA contribute to virulence in a murine model of invasive pulmonary aspergillosis. (A) Percent survival of mice infected with 2 × 10^6^ conidia of the Δ*srcA*/Δ*pmrA* mutant or the KU80 control strain. Groups of 12 male CF-1 mice were immunosuppressed with a single dose of triamcinolone acetonide 1 day before the infection. The mutant showed significantly attenuated virulence (***, *P < *0.001 [log rank test]). (B) Histopathological analysis of lungs from mice that were treated as described for panel A in a separate experiment and sacrificed 3 days after infection. Sections were stained with Gomori’s methenamine silver (GMS) or hematoxylin and eosin (HE). Bars: 100 μm.

Since the A. fumigatus UPR is necessary for virulence (13, 14), and we demonstrate here that *srcA* and *pmrA* are novel UPR targets, we assessed the contribution of these genes to traits that are linked to disease pathogenesis. The Δ*srcA*/Δ*pmrA* mutant caused less damage to a monolayer of the A549 pulmonary epithelial cell line than either the parental strain or the two single gene deletion mutants (Fig. 6A). In addition, the double deletion mutant was unable to effectively use lung tissue as a substrate (Fig. 6B). The Δ*pmrA* mutant was previously shown to cause the same level of mortality as wild-type A. fumigatus in a mouse infection model (34). We also found no virulence defect in this strain using Galleria mellonella as an alternative animal infection model (Fig. S7A). Similarly, we found that the Δ*srcA* mutant retained full virulence in two immunologically distinct mouse models of invasive aspergillosis: a triamcinolone acetonide (TA)-induced steroid immunosuppression model and a cyclophosphamide/TA-induced leukopenic model (Fig. S7B). In contrast, the Δ*srcA*/Δ*pmrA* mutant had reduced virulence in both male and female mice immunosuppressed by TA, with at least 50% survival in both groups after 2 weeks (Fig. 7A; see also Fig. S7C). Histopathological analysis of lung tissue on day 3 postinoculation revealed decreased fungal growth and reduced peribronchiolar inflammation in the Δ*srcA*/Δ*pmrA* mutant-infected mice (Fig. 7B). Comparable findings were made in the insect model: the Δ*srcA*/Δ*pmrA* mutant was avirulent at a low inoculum of conidia and was attenuated when larvae were infected with a high amount of conidia (Fig. S7A). Reconstitution of the *pmrA* gene into the Δ*srcA*/Δ*pmrA* mutant fully rescued virulence (Fig. S7A). Together, these findings demonstrate that SrcA and PmrA are individually dispensable in the host environment but that their functions are jointly required to support the expression of virulence attributes that are needed during infection.

10.1128/mBio.01060-20.7 FIG S7 SrcA and PmrA jointly contribute to virulence in different models of invasive aspergillosis. (A) Insect model of fungal infection. Groups of at least 25 larvae of the greater wax moth Galleria mellonella were infected with 2 × 10^5^ (left) or 1 × 10^6^ (right) conidia from the indicated strains and incubated for 7 days at 37°C. Only the Δ*srcA*/Δ*pmrA* mutant showed significantly attenuated virulence relative to the KU80 parental strain (****, *P < *0.0001 [log rank test]). (B) The Δ*srcA* mutant retained virulence in two murine models of invasive pulmonary aspergillosis. (Left) Steroid model in female CF-1 mice treated with a single dose of triamcinolone at day −1 and infected with 2 × 10^6^ conidia from the indicated strains (12 mice per group). (Right) Leukopenic model using two doses of cyclophosphamide on days −2 and +3 and triamcinolone acetonide on days −1 and +6. Groups of 12 female CF-1 mice were infected with 1 × 10^6^ conidia per mouse per condition. (C) Steroid model of invasive pulmonary aspergillosis using the same model as described in the Fig. 6A legend, except for the use of female mice (12 per group). The Δ*srcA*/Δ*pmrA* mutant shows significantly attenuated virulence (***, *P < *0.001 [log rank test]). Download FIG S7, JPG file, 1.7 MB. Copyright © 2020 Weichert et al. 2020 Weichert et al. This content is distributed under the terms of the Creative Commons Attribution 4.0 International license.

### Loss of HacA disrupts Ca^2+^ homeostasis.

**FIG 8:**
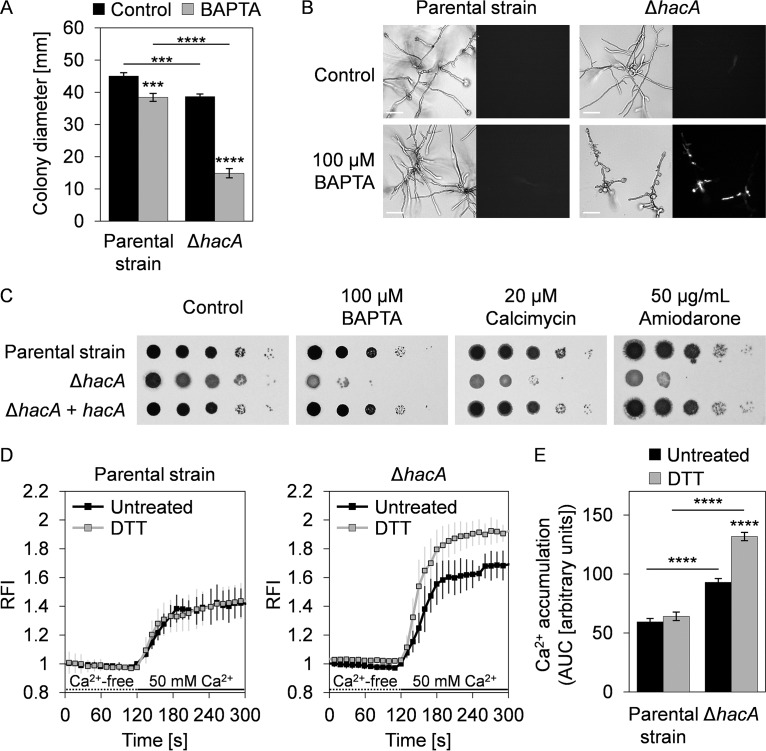
Loss of HacA disrupts Ca^2+^ homeostasis. (A) Quantification of radial growth of mycelia from the KU70 parental strain and the Δ*hacA* mutant grown for 7 days at 37°C on AMM plates with and without BAPTA (100 μM). Values represent means ± SD of results from triplicate plates (***, *P < *0.001; ****, *P < *0.0001 [ANOVA with Tukey’s *post hoc* test]). (B) Hyphal morphology of the indicated strains grown in liquid AMM with and without BAPTA for 24 h at 37°C. Following growth, the cultures were stained with propidium iodide (PI) and inspected by bright-field microscopy (left panels) and fluorescence microscopy (right panels). Fluorescence in the PI-stained cultures reveals death of hyphal compartments. Bars: 50 μm. (C) Serial 10-fold dilutions of conidia from the indicated strains were spotted onto AMM plates containing BAPTA, calcimycin, or amiodarone and incubated for 2 days at 37°C. (D) Analysis of Ca^2+^ signatures in the KU70 parental strain and the Δ*hacA* mutant expressing the cytosolic fluorescent Ca^2+^ reporter GCaMP5. After growth in liquid YG medium, germlings were treated for 1 h with 1 mM DTT or left untreated, followed by sequential perfusion with Ca^2+^-free Ringer’s solution (dotted lines) and buffer containing 50 mM Ca^2+^ (solid lines). Curves show the normalized relative fluorescence intensities (RFI) over time from 5 to 7 independent experiments per strain and condition (means ± SD). For each curve, RFI values were calculated by using the average fluorescence intensity at 120 s from the untreated samples of each strain as the reference. (E) The accumulation of cytosolic Ca^2+^ was quantified from the area under the curve (AUC) based on the graphs shown in panel D during the perfusion with 50 mM Ca^2+^, setting the RFI at 120 s for each curve as the lower cutoff. AUC values represent means ± SEM (****, *P < *0.0001 [one-way ANOVA with Tukey’s *post hoc* test]).

The ability of the UPR to regulate the expression of genes encoding ER/Golgi Ca^2+^ pumps in proportion to demand suggested that blocking this pathway would be deleterious under conditions of Ca^2+^ stress. To test this, we examined the A. fumigatus Δ*hacA* mutant for susceptibility to agents that disrupt intracellular Ca^2+^ balance. Concentrations of BAPTA that had minimal effects on the parental strain inhibited the growth of the Δ*hacA* mutant, which was associated with death of hyphal compartments (Fig. 8A and B). Similarly, the Δ*hacA* mutant was hypersensitive to calcimycin and amiodarone (Fig. 8C), which are compounds that use distinct mechanisms to disrupt cytosolic and store-related Ca^2+^ levels (40, 41). To further demonstrate that the canonical UPR impacts Ca^2+^ homeostasis, we examined cytoplasmic Ca^2+^ levels by expressing an optimized variant of the fluorescent genetically encoded Ca^2+^ indicator GCaMP5 (42). We found that a sustained increase in the level of cytosolic Ca^2+^ was a normal response of the parental strain to an increase in extracellular Ca^2+^ (Fig. 8D). The accumulation of cytosolic Ca^2+^ in the presence of high levels of extracellular Ca^2+^ was also evident in the Δ*hacA* strain, but the magnitude of the influx was higher, consistent with deregulation of mechanisms that maintain cytosolic Ca^2+^ within a normal range. Moreover, although pretreatment with DTT to induce ER stress had no effect on cytoplasmic Ca^2+^ levels in the parental strain, it was associated with an even greater influx in the Δ*hacA* mutant (Fig. 8D and E). These data indicate that loss of HacA impairs the ability of the fungus to maintain cytosolic Ca^2+^ within normal levels and that the effect is aggravated under ER stress conditions, possibly due to the inability to regulate ER/Golgi Ca^2+^ pumps.

### Loss of HacA and its targets SrcA and PmrA increases susceptibility to calcineurin pathway inhibition.

**FIG 9:**
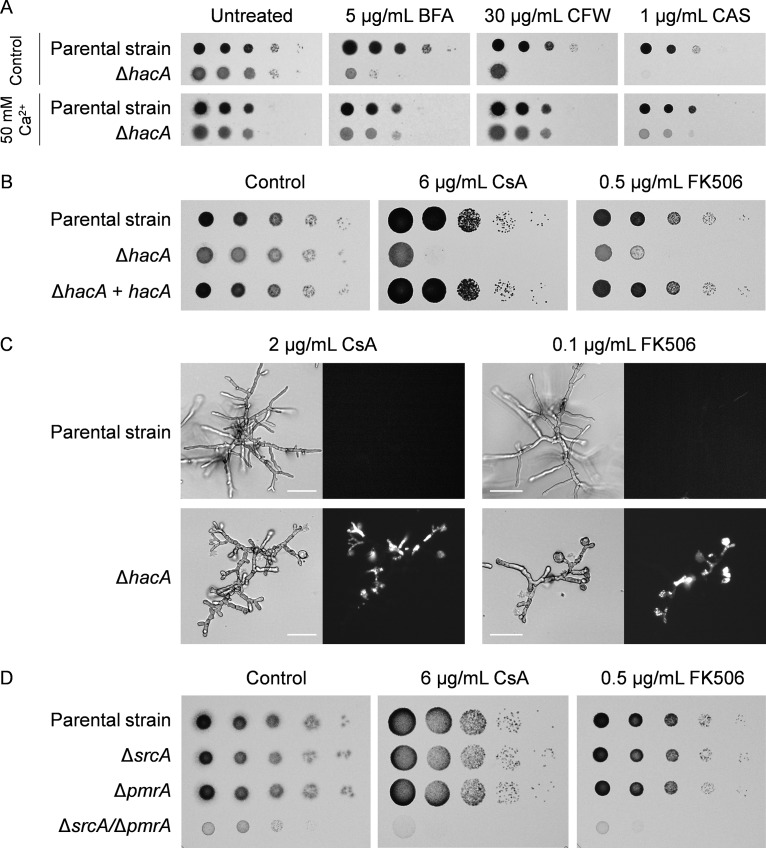
SrcA, PmrA, and the canonical UPR protect against calcineurin inhibition. (A) Serial 10-fold dilutions of conidia from the KU70 parental strain and the Δ*hacA* mutant were incubated for 2 days at 37°C on AMM plates without or with supplementation with Ca^2+^ and in the presence or absence of brefeldin A (BFA), calcofluor white (CFW), or caspofungin (CAS). (B) The indicated strains were incubated for 2 days at 37°C on AMM plates in the presence of cyclosporine (CsA) or FK506. (C) Bright-field and PI-stained fluorescent images of hyphae after 24 h at 37°C in liquid AMM containing CsA or FK506. No fluorescence was observed in untreated controls (see Fig. 8B). Bars: 50 μm. (D) Serial 10-fold dilutions of conidia from mutants lacking *srcA* and/or *pmrA* and the KU80 parental strain were spotted onto AMM in the presence or absence of FK506 or CsA and incubated for 2 days at 37°C.

Deletion of *hacA* in A. fumigatus, or of its homologs in other fungal species, is known to cause hypersensitivity to ER and cell wall stress agents (25). Interestingly, we found that this hypersensitivity was rescued by increasing the availability of external Ca^2+^ (Fig. 9A). Since Ca^2+^ acts as a second messenger in the cytosol (43), we hypothesized that the activation of a Ca^2+^-responsive signal transduction pathway in the Δ*hacA* mutant might be compensating for the lack of UPR signaling. One of the major pathways that controls adaptive intracellular Ca^2+^ signaling in fungi is directed by the Ca^2+^/calmodulin-activated protein phosphatase calcineurin, which can be inhibited pharmacologically with cyclosporine (CsA) or FK506 (44, 45). Both of these calcineurin inhibitors showed potent antifungal activity against the Δ*hacA* mutant (Fig. 9B). Propidium iodide staining revealed that these compounds induced loss of viability in hyphal compartments (Fig. 9C), indicating that this mutant relies heavily on calcineurin intervention for survival. Similarly, we found that the Δ*srcA*/Δ*pmrA* mutant was unable to grow at concentrations of these drugs that were subinhibitory for control strains, including the two single deletion mutants (Fig. 9D). These findings imply that the absence of either the canonical UPR or of its two downstream targets SrcA and PmrA creates a defect in Ca^2+^ homeostasis that increases dependency on calcineurin-dependent signaling.

## DISCUSSION

Previous studies have shown that loss of Ca^2+^ homeostasis causes ER stress, thereby triggering the UPR (46, 47). The best-understood function of the UPR in mitigating ER stress is the transcriptional upregulation of the protein folding and modification machinery, which comprises folding enzymes, chaperones, and glycosylating enzymes (12, 25). Since many of these proteins require Ca^2+^ as a cofactor (28), an increase in their levels needs to be coordinated with an adequate supply of these ions. One of the major mechanisms for transporting Ca^2+^ into the ER of higher eukaryotes involves the SERCA family of P-type Ca^2+^-ATPases (28). Here, we demonstrate that the transcription of the A. fumigatus *srcA* gene, encoding the only member of the SERCA family of P-type Ca^2+^-ATPases in this fungal species, is upregulated by ER stress in a manner that is dependent on the UPR transcription factor HacA. In addition, we found that *srcA* induction was paired with HacA-dependent upregulation of the *pmrA* gene (Fig. 1B), encoding a member of the SPCA family of Golgi Ca^2+^-ATPases (34).

Deletion of either *srcA* or *pmrA* was not associated with increased expression of the other gene (see Fig. S2B in the supplemental material), indicating the absence of compensatory transcription. Interestingly, deletion of the homologs of SrcA and PmrA in the filamentous fungal insect pathogen Beauveria bassiana was associated with induction of three other P-type Ca^2+^-ATPases of the PMCA family (48). This was not the case in A. fumigatus, since the Δ*srcA*/Δ*pmrA* mutant showed no compensatory upregulation of *pmcA*, *pmcB*, or *pmcC* (Fig. S2C), signifying fundamental differences in gene regulation between these fungal species. Interestingly, the A. fumigatus *pmcA/B/C* genes were induced by Ca^2+^, as previously reported in other species (29, 49, 50), but were not subject to HacA-dependent induction during ER stress (Fig. S3A and B). This contrasted with the results seen with the *srcA* and *pmrA* genes, which were unresponsive to Ca^2+^ (Fig. S3B). Together, these findings suggest that A. fumigatus P-type Ca^2+^-ATPases can be minimally divided into two functional classes: one represented by *srcA* and *pmrA*, which are induced by HacA during ER stress but not by external Ca^2+^, and the other represented by the *pmcA/B/C* genes, which are not under HacA control but are subject to upregulation by external Ca^2+^. This implies that the UPR drives Ca^2+^ influx into the ER/Golgi compartments in proportion to demand, rather than globally upregulating all Ca^2+^ pumps as a means to remove excess cytosolic Ca^2+^ that may accumulate under stress conditions. Together, these findings suggest that the expression of genes encoding Ca^2+^-ATPases of the secretory pathway is titrated by the canonical UPR, providing a mechanism to coordinate ER/Golgi Ca^2+^ availability with the rising levels of Ca^2+^-dependent chaperones and processing enzymes that are concomitantly induced by UPR activation.

The A. fumigatus *srcA* gene was dispensable for normal vegetative growth on rich and defined medium. This result is similar to what has been described previously for the corresponding mutants of SERCA homologs (called Eca1/NCA-1) in the corn smut fungus Ustilago maydis, the human-pathogenic yeast Cryptococcus neoformans, and the saprophyte Neurospora crassa (30, 51, 52) but differs from what has been described previously for B. bassiana, where the growth of the Δ*eca1* mutant was impaired on multiple carbon and nitrogen substrates (48). While the A. fumigatus Δ*pmrA* mutant showed a moderate growth defect, the absence of both *srcA* and *pmrA* severely reduced colony formation and almost completely blocked conidiation, similarly to findings in N. crassa (53). Since additional Ca^2+^ in the medium restored growth and sporulation, this implies that other mechanisms of Ca^2+^ import (36) can partially compensate for the loss of SrcA and PmrA. However, our data indicate that A. fumigatus relies heavily on SrcA and PmrA to provide sufficient Ca^2+^ import into the secretory pathway to support normal hyphal foraging and conidial development.

The induction of *srcA* and *pmrA* by ER stress implies that these genes contribute to ER/Golgi homeostasis. Evidence to support a role for SERCA-type Ca^2+^-ATPases in ER stress has been found in U. maydis, B. bassiana, and C. neoformans (30, 48, 51). Surprisingly, we found no increase in susceptibility of A. fumigatus Δ*srcA* to treatment with DTT, BFA, or TM, indicating differences in the levels of dependency on SERCA homologs among species. However, the Δ*srcA*/Δ*pmrA* mutant was hypersensitive to ER stress conditions and showed increased levels of steady-state expression of the UPR target gene *pdiA*, encoding a Ca^2+^-dependent protein disulfide isomerase (Fig. 3A and D). This suggests that ER stress levels are elevated in the Δ*srcA*/Δ*pmrA* mutant, possibly due to impaired ER/Golgi Ca^2+^ import from the cytoplasm. Consistent with this, expression of the GCaMP5 reporter revealed increased baseline levels of cytosolic Ca^2+^ in the Δ*srcA*/Δ*pmrA* mutant under conditions of ER stress (Fig. S8A and B). Thus, despite the ability of this strain to induce the canonical UPR during ER stress (Fig. 3C), we speculate that a reduction in ER/Golgi Ca^2+^ levels would impair Ca^2+^-dependent protein folding processes, leading to the hypersensitivity of this strain to ER stress. In contrast, consistent with functional redundancy between SrcA and PmrA in maintaining Ca^2+^ homeostasis in the interconnected ER and Golgi compartments (10), the single mutants did not show an increase in baseline levels of cytosolic Ca^2+^ (Fig. S8B). While all of these GCaMP5-expressing Ca^2+^ pump mutants accumulated cytosolic Ca^2+^ in response to high levels of extracellular Ca^2+^, we noted that this increase was significantly attenuated in the Δ*srcA*/Δ*pmrA* mutant in the presence or absence of ER stress (Fig. S8C), supporting further the conclusion that this strain is defective in Ca^2+^ homeostasis. However, since the cytosolic Ca^2+^ signatures of these Ca^2+^ pump mutants differed from those of the UPR Δ*hacA* mutant (Fig. 8D and E), we speculate that the UPR may control additional, as-yet-unidentified factors involved in Ca^2+^ homeostasis when both SrcA and PmrA are absent. This might also account for the attenuated accumulation of cytosolic Ca^2+^ in the Δ*srcA* mutant during ER stress (Fig. S8C), underscoring an important role of SrcA in ER stress adaptation.

10.1128/mBio.01060-20.8 FIG S8 Loss of SrcA and PmrA alters cytosolic Ca^2+^ levels. (A) Ca^2+^ signatures in the KU80 parental strain and mutants with deletions of *srcA* and/or *pmrA* expressing GCaMP5. After growth in YG medium in the presence or absence of DTT (1 mM for 1 h prior to analysis), the cultures were initially perfused with Ca^2+^-free buffer (dotted lines), followed by perfusion with 50 mM Ca^2+^ (solid lines). Curves show the normalized relative fluorescence intensities (RFI) over time from 3 to 7 independent experiments per strain and condition (means ± SD). For each curve, the RFI values were calculated by using the average fluorescence intensity measured at 120 s for the untreated samples of each strain as the reference. (B) Baseline cytosolic Ca^2+^ levels of the indicated strains were obtained from the curves shown in panel A based on the RFI after 60 s of perfusion with Ca^2+^-free Ringer’s solution. Values represent means ± SD of results from all experiments per strain and condition (****, *P < *0.0001 [unpaired, two-tailed *t* tests]). (C) The accumulation of cytosolic Ca^2+^ was quantified from the area under the curve (AUC) in the graphs shown in panel A corresponding to the perfusion with 50 mM Ca^2+^, setting the RFI at 120 s for each curve as the lower cutoff. AUC values represent means ± SEM of results from all experiments (**, *P < *0.01; ***, *P < *0.001 [one-way ANOVA with Tukey’s *post hoc* test]). Download FIG S8, JPG file, 1.6 MB. Copyright © 2020 Weichert et al. 2020 Weichert et al. This content is distributed under the terms of the Creative Commons Attribution 4.0 International license.

The Δ*pmrA* mutant showed increased sensitivity to cell wall perturbation by CFW and CR. However, deletion of *srcA* did not cause an increase in sensitivity to either compound, suggesting that PmrA has the dominant role in supporting cell wall stress homeostasis. Biochemical analysis of the cell wall monosaccharide composition revealed alterations in all of the mutants, the most striking of which was a severe loss of mannose in the Δ*srcA*/Δ*pmrA* mutant (Fig. 5B). Since galactomannan is the major destination of mannose in the cell wall of A. fumigatus (54), it is remarkable that mutants of this species that are either deficient in Golgi-resident Ktr mannosyltransferases or unable to insert galactomannan into the β-(1,3)-glucan-chitin core of the cell wall display colony morphologies reminiscent of the Δ*srcA*/Δ*pmrA* mutant (55, 56). Since these mutants depleted in galactomannan also share hypersensitivity to CFW and CR with the Δ*srcA*/Δ*pmrA* mutant, we speculate that the Ca^2+^ defect created by the absence of SrcA and PmrA inhibits the activity of Ktr and/or Dfg proteins, resulting in abnormalities in the biosynthesis and/or distribution of cell wall mannans.

Deletion of the *eca1* gene encoding a SERCA homolog in C. neoformans attenuated virulence in a G. mellonella model at 37°C but not at 30°C, suggesting that the reduction in pathogenicity could be attributed to a loss of thermotolerance (51). A similar reduction in thermotolerance was reported previously for the corresponding mutant in U. maydis (30), as well as for the A. fumigatus Δ*srcA* mutant in this study (Fig. 3B; see also Fig. S4C), supporting the idea of a conserved role for fungal SERCA homologs in growth at elevated temperatures. However, the A. fumigatus Δ*srcA* mutant was temperature sensitive at 45°C rather than at 37°C and was therefore unaffected in virulence (Fig. S7B). In contrast, the Δ*srcA*/Δ*pmrA* mutant showed reduced virulence in both mouse and insect infection models. The severe growth defect of the Δ*srcA*/Δ*pmrA* mutant likely accounts for much of this reduced virulence capacity. However, it is also possible that changes in cell wall structure and composition that occur *in vivo* may be different from those that occur *in vitro*, which could affect inflammatory responses or colony morphology and thus would also impact virulence (39, 54, 57). Together, these findings indicate that the UPR targets SrcA and PmrA are jointly required to support the virulence of A. fumigatus, providing further support for the idea of UPR as a regulatory hub for fungal pathogenicity.

Unlike S. cerevisiae, where deletion of *hac1* triggered no increase in sensitivity to Ca^2+^ chelation (58), the corresponding mutant in A. fumigatus was hypersensitive to BAPTA (Fig. 8). This suggests that the inability of the Δ*hacA* mutant to transcriptionally regulate *srcA* and *pmrA* aggravates the progressive depletion of internal Ca^2+^ stores, such as the ER and Golgi compartments, caused by Ca^2+^ starvation. Conversely, we found that supplementing the medium with extra Ca^2+^, which raises cytosolic Ca^2+^ levels (Fig. 8D), rescued the Δ*hacA* mutant from ER and cell wall stress (Fig. 9A). Somewhat paradoxically, treatment with drugs that are also known to increase levels of cytosolic Ca^2+^ (amiodarone and calcimycin) was toxic to the UPR mutant (Fig. 8C). This likely reflects the additional ability of calcimycin and amiodarone to trigger Ca^2+^ release from the ER, Golgi, and vacuolar compartments (40, 41), resulting in a broader disruption of Ca^2+^ homeostasis than that seen with Ca^2+^ supplementation alone. Taken together, these findings are consistent with a model in which the Δ*hacA* mutant is less capable of buffering fluctuations in cytosolic Ca^2+^ than the parental strain. Moreover, the beneficial effect of extracellular Ca^2+^ on the Δ*hacA* mutant suggests that some of the phenotypes associated with loss of UPR function in A. fumigatus may be attributable, at least in part, to insufficient Ca^2+^ in the secretory pathway, originating in the inability to upregulate *srcA* and *pmrA* genes under ER stress conditions. A second possibility, which is not mutually exclusive with the first, is that supplemented extracellular Ca^2+^ compensates for the loss of HacA by activating the Ca^2+^-responsive calcineurin signaling pathway, which also mediates stress adaptation in fungi (44). In support of this, we found that calcineurin inhibitors showed increased potency against the Δ*hacA* mutant (Fig. 9B and C). Since calcineurin is considered a strong target for emerging antifungal therapy (59), this raises the possibility that future approaches designed to interrupt the UPR could be harnessed to enhance the efficacy of calcineurin inhibitors in combination therapy.

## MATERIALS AND METHODS

### Strains and growth conditions.

All the strains of A. fumigatus used in this study are listed in Table S1 in the supplemental material. Unless otherwise stated, experiments were performed in liquid *Aspergillus* minimal medium (AMM) {1% (wt/vol) d-glucose, 1% (vol/vol) NH_4_ tartrate, 2% (vol/vol) salt solution [2.6% (wt/vol) KCl, 2.6% (wt/vol) MgSO_4_ heptahydrate, 7.6% (wt/vol) KH_2_PO_4_, 5% (vol/vol) trace-element solution]} or AMM plates with 0.8% (wt/vol) UltraPure agarose (Invitrogen). Conidia were harvested from mycelia grown for 1 week at 37°C on AMM plates containing 1.2 M sorbitol (OSM [osmotically stabilized medium]). Since the Δ*srcA*/Δ*pmrA* mutant conidiated poorly on AMM, conidia were obtained from rich medium (inhibitory mold agar [IMA]) (Becton, Dickinson) supplemented with 50 mM CaCl_2_ and were incubated for up to 10 days at 37°C. These conidia were harvested in sterile phosphate-buffered saline (PBS) supplemented with 0.1% (vol/vol) Tween 20 and were sequentially passed through cell strainers with a pore size of 40 μm (Fisher Scientific) or 10 μm (pluriSelect) prior to washing with sterile distilled water. Radial growth was measured by spotting 5 × 10^3^ conidia in a 5-μl droplet onto the center of AMM or IMA plates and monitoring colony diameter with time. Stress sensitivities were assessed by spotting serial 10-fold dilutions of conidia (10^5^ to 10 spores in droplets of 5 μl) onto AMM plates containing a stress agent. The chemicals used to induce stress included dithiothreitol (Thermo Scientific), tunicamycin (Cayman Chemical), brefeldin A (Enzo), calcofluor white (Sigma), Congo red (Sigma), hygromycin B (RPI), BAPTA (Invitrogen), calcimycin [A23187] (Sigma), amiodarone (Sigma), cyclosporine (InvivoGen), and FK506 (InvivoGen).

10.1128/mBio.01060-20.9 TABLE S1 Strains of Aspergillus fumigatus used in this study. Download Table S1, DOCX file, 0.02 MB. Copyright © 2020 Weichert et al. 2020 Weichert et al. This content is distributed under the terms of the Creative Commons Attribution 4.0 International license.

### Genetic modifications.

Gene deletion and complementation and *in situ* tagging were achieved by homologous recombination with the target locus using 5′ and 3′ flanking regions of about 1 kb that were PCR amplified from genomic DNA. Recipient strains for transformations contained a deletion of the *akuA*^KU70^ or *akuB*^KU80^ gene for efficient site-specific integration as previously described (60, 61). A recyclable marker module (MM) was used for selection (62) and contained the hygromycin B phosphotransferase (*hph*) gene or chlorimuron-ethyl resistance (*cme*^R^) gene (63), as well as the beta-recombinase (β-*rec*) gene under the control of a xylose-responsive promoter (P*xyl*), all flanked by two *six* sites for β-Rec-mediated self-excision of the MM. The MM was PCR amplified with primer pair 1053/1054 (Table S2) from vectors pSK529 (*hph*-β-*rec*; gift from Sven Krappmann) and p680 (*cme*^R^-β-*rec*; gift from Jean-Paul Latgé). The pUC19L backbone for selection in bacteria was PCR amplified from vector pUC19 with primers 1061/1062 containing restriction sites for linearization prior to transformation.

10.1128/mBio.01060-20.10 TABLE S2 List of oligonucleotides used in this study. Download Table S2, DOCX file, 0.03 MB. Copyright © 2020 Weichert et al. 2020 Weichert et al. This content is distributed under the terms of the Creative Commons Attribution 4.0 International license.

To tag the *srcA* gene with *egfp in situ*, the left arm spanning the coding sequence of the *srcA* gene was PCR amplified with primers 1341/1342, the right arm was amplified with primers 1106/1107, and the *egfp* sequence was amplified from plasmid pEGFP-N1 with primers 1125/1119. The fragments were assembled with *hph*-β-*rec* and pUC19L using a GeneArt seamless cloning and assembly kit (Thermo Fisher), creating plasmid p704. Site-specific integration of the construct was confirmed by PCR (data not shown).

To delete *srcA*, the left and right arms flanking the open reading frame of the *srcA* gene were PCR amplified with primers 1057/1049 and 1058/1050, respectively, and assembled with *hph*-β-*rec* and pUC19L as described above, resulting in vector p670. Similarly, the p691 knockout plasmid used for the deletion of *pmrA* was generated with primers 1160/1161 and 1162/1163 to obtain the flanking regions, which were assembled with *cme*^R^-β-*rec* and pUC19L. Transformations were performed as described previously (64). For hygromycin B selection, protoplasts were plated onto OSM plates and incubated overnight at room temperature prior to overlaying with top agar containing hygromycin B to reach a final concentration of 150 μg/ml. For chlorimuron-ethyl selection, transformants were plated directly onto OSM plates supplemented with 50 μg/ml chlorimuron ethyl (Fisher Scientific). Monoconidial transformants were passaged onto AMM plates containing 1% (wt/vol) xylose as the sole carbon source to excise the MM. The Δ*srcA*/Δ*pmrA* mutant was generated by transforming protoplasts of Δ*srcA* with p691 as described above. Confirmation of all genotypes was performed by PCR (see Fig. S4A in the supplemental material; see also Fig. S5A) (Table S2).

For complementation of deletion mutants, the *srcA* and *pmrA* genes were amplified from genomic DNA with primers 1003/1006 and 1311/1312, respectively. The *srcA* gene was cloned into PCR4 Blunt TOPO vector to generate plasmid p686. For *pmrA* complementation, a 1.9-kb intergenic region (IR) from chromosome 1 (between the loci Afu1g04960 and Afu1g04970) was PCR amplified with primers 1077/1078 and cloned into the *pmrA* vector (p701), allowing site-specific targeting to the IR after linearizing with BsaBI. Complementation vectors p686 and p701 were cotransformed with selectable marker vector p680 (linearized with FspI) in at a stochiometric ratio of 10 to 1. To complement the Δ*srcA*/Δ*pmrA* mutant, protoplasts generated from 50-ml yeast extract-glucose (YG) cultures grown for 24 h at 30°C and 100 rpm were transformed with linearized p701 and the *hph*-encoding vector pAN7.1. Confirmation of complemented genotypes was performed by PCR analysis (Fig. S4; see also Fig. S5).

For expression of the genetically encoded Ca^2+^ indicator GCaMP5, the construct P*_C.h.gpd1_*-*gcamp5*-T*_N.c.β-tubulin_*, which was PCR amplified with primers 1079/1080 from pSK3042 (a kind gift from Seogchan Kang [unpublished data]), was first subjected to *in vitro* assembly with pUC19L and the IR sequence as described above, resulting in vector p673. The *hph*-β-*rec* marker module was then inserted into a HindIII site in p673, resulting in p675. Since the Δ*hacA* mutant (strain 144; Table S1) contains a nonrecyclable hygromycin B marker, a second Δ*hacA* mutant was created using the self-excising *hph*-β-*rec* marker module in a manner analogous to the method used for deletion of the *srcA* gene (primer pair 1055/1047 and primer pair 1048/1056). The resulting p669 plasmid was linearized with FspI followed by transformation of conidia by electroporation as previously described (65). After purification of hygromycin-resistant colonies under conditions of selective pressure and passaging onto xylose-containing medium to induce marker module excision, the new Δ*hacA* transformants were verified by analysis of sensitivity to hygromycin B and by PCR (data not shown). The resulting Δ*hacA* strain, named strain 467 (Table S1), showed hypersensitivity to ER-, cell wall-, and Ca^2+^-related stress agents similar to that shown by the original strain, strain 144 (data not shown). The p675 GCaMP5 construct was linearized with EcoRV prior to transformation into the KU70, Δ*hacA* (strain 467), KU80, Δ*srcA*, and Δ*pmrA* strains. To create a Δ*srcA*/Δ*pmrA* mutant expressing GCaMP5, the *pmrA* gene was deleted in the Δ*srcA* GCaMP5 strain as described above. Site-specific integration and expression of the GCaMP5 construct were confirmed by PCR analysis and Western blotting using an anti-green fluorescent protein (anti-GFP) antibody (data not shown).

### Quantitative reverse transcription PCR (RT-qPCR) analysis.

Unless otherwise stated, flasks containing 50 ml of liquid YG medium were inoculated with 1 × 10^6^ conidia/ml and incubated for 16 h at 37°C and 200 rpm, followed by treatment with DTT for 1 h. RNA was extracted from biomass ground using liquid nitrogen and an RNAzol RT column kit (MRC, Inc.). After treatment of the extracts with DNase I (Roche), cDNA was synthesized using iScript Reverse Transcription Supermix for RT-qPCR (Bio-Rad). Using a StepOne real-time PCR system (Applied Biosystems), the reaction mixtures were prepared as triplicates with 1 μg of cDNA, 500 nM concentrations of the gene-specific primers listed in Table S2 (200 nM for the housekeeping gene *18S rRNA*), and iTaq Universal SYBR green Supermix (Bio-Rad). Amplification parameters were set to 20 s at 95°C, 40 cycles of 3 s at 95°C, and 30 s at 60°C (with the exception of 20 s at 66°C for the *hacA^u^*^/^*^i^* primers). Melting curves were generated to verify the specificity of the reactions. Fold changes in transcript levels were calculated from threshold cycle (ΔΔ*C_T_*) values in comparison to samples from the parental strain or untreated controls. Primer efficiencies (between 95 and 105%) were determined with cDNA standard curves. All experiments were repeated at least once with cDNA obtained from independent cultures.

### Bright-field and fluorescence microscopy.

About 1 × 10^3^ conidia were incubated overnight in liquid medium on glass or polypropylene carriers. Differential interference contrast, bright-field, and fluorescence images were captured using Olympus BH-2, IX71, and BX51 microscopes and were adjusted for brightness and contrast with ImageJ. To stain dead cells, cultures were incubated for 5 min with 10 μM propidium iodide (PI; Cayman Chemicals) prior to imaging.

### Transmission electron microscopy (TEM).

Overnight cultures in liquid YG medium were fixed for at least 2 h at 4°C in 0.1 M cacodylate buffer (pH 7.4) containing 2% (wt/vol) glutaraldehyde and 2% (wt/vol) paraformaldehyde prior to treatment for 2 h in 1% (wt/vol) osmium tetroxide. After rinses with buffer, specimens were stepwise dehydrated in graded alcoholic solutions and embedded in LX112 resin. Thin sections were stained with uranyl acetate followed by lead citrate. Digital images were acquired on a JEOL 1230 transmission electron microscope equipped with an AMT Advantage Plus digital camera (2,000 by 2,000 pixels) at 80 kV. To determine cell wall thickness, the average width of the electron-lucent region (obtained from four equally spaced measurements per hyphal cross section) was determined for 10 hyphae per strain using ImageJ.

### Analysis of cell wall monosaccharide composition.

Flasks containing 50 ml of liquid YPD medium were inoculated with 1 × 10^8^ conidia and incubated for 40 h at 37°C with constant shaking (150 rpm). Mycelia were collected by filtration and subjected to cell wall carbohydrate analysis as previously described (14). The total monosaccharide composition obtained from the alkali-soluble (AS) and alkali-insoluble (AI) fractions was calculated for each strain from three independent cultures.

### Epithelial cell damage assay.

The extent of damage to A549 pulmonary epithelial cells was measured using a standard ^51^Cr release assay as detailed previously (66). In brief, after the epithelial cells were loaded with ^51^Cr in 24-well tissue culture plates, they were infected in F-12 K medium with 5 × 10^5^ conidia from the KU80 parental strain or the mutants lacking *srcA* and/or *pmrA* and incubated for 24 h at 37°C in 5% (vol/vol) CO_2_. For each fungal strain, the percentage of specific release of ^51^Cr from the epithelial cells was calculated from triplicates in three independent experiments.

### Animal models of invasive aspergillosis.

For the steroid model, groups of 12 male (26 to 33 g) or female (24 to 30 g) CF-1 outbred mice (Charles River) were immunosuppressed by subcutaneous injection with a single dose of triamcinolone acetonide (TA) (40 mg/kg of body weight) on day −1. The next day, the mice were anesthetized with 3.5% isoflurane and intranasally infected with 20 μl of saline solution containing 2 × 10^6^ conidia or with sterile saline solution. Survival was monitored for 2 weeks. For the leukopenic model, female CF-1 mice (25 to 28 g) were immunosuppressed by intraperitoneal injection of cyclophosphamide (150 mg/kg) on days −2 and +3 and by subcutaneous injection of TA (40 mg/kg) on days −1 and +6. For histopathological analysis of murine lung tissues, male CF-1 mice in the steroid model were infected as described above and sacrificed on day 3 postinfection. After the lungs were fixed for 48 h in 10% neutral buffered formalin solution (Sigma), the samples were dehydrated, embedded in paraffin, sectioned at 5 μm, stained with Gomori’s methenamine silver (GMS) or hematoxylin and eosin (HE), and imaged with an Olympus BX51 microscope. All conidial stocks used for inoculation were plated to verify viability.

For the insect model, groups of at least 25 similarly sized larvae of G. mellonella were infected in the right last pro-leg with 20 μl of PBS containing 2 × 10^5^ or 1 × 10^6^ conidia using U-100 insulin syringes (28 G × 1/2 in; Becton, Dickinson). Larvae were kept for 7 days at 37°C in the dark and monitored daily. Larvae were scored as dead upon displaying dark-brown pigmentation and loss of motility.

### Ethics statement.

The mouse studies were performed in agreement with the recommendations in the Guide for the Care and Use of Laboratory Animals of the National Research Council. Our animal use protocol was approved by the Institutional Animal Care and Use Committee (IACUC) at the University of Cincinnati.

### Analysis of Ca^2+^ signatures with GCaMP5.

Plates (30 mm in diameter) containing 3 ml of liquid YG medium and a glass coverslip were inoculated with about 1 × 10^3^ conidia from GCaMP5-expressing strains and incubated overnight at room temperature. After the cultures were shifted to 37°C for 3.5 to 4.5 h (except for the direct incubation of the Δ*srcA*/Δ*pmrA* mutant at 37°C for 13 to 15 h) and optionally treated for 1 h with 1 mM DTT prior to analysis, coverslips were placed into imaging chambers and perfused for at least 2 min at a rate of about 3 ml/min with Ca^2+^-free Ringer’s solution supplemented with 100 μM EGTA using a dedicated workstation vacuum system (Warner Instruments), followed by perfusion with a solution containing 50 mM CaCl_2_. Ca^2+^ signatures composed of up to 20 germlings per sample were recorded over time on a Nikon TMS-F microscope equipped with a UV-F 40× glycerin immersion lens objective (numerical aperture [NA], 1.3), a xenon light source, and a Scout scA640-74f camera (Basler Vision Technologies) coupled to a PC using the InCyt-Im1 image analysis program (Intracellular Imaging). To account for differences in GCaMP5 expression and cell sizes between strains, the fluorescence intensities occurring over time were normalized for each strain relative to the fluorescence value of the untreated control prior to perfusion with Ca^2+^. The average Ca^2+^ signatures (means ± standard deviations [SD]) of the untreated and DTT-treated cultures were calculated from at least three independent experiments per strain and condition. Area under the curve (AUC) values were generated as means ± standard errors of the means (SEM) with GraphPad Prism (V. 8.3.1).

### Statistical analysis.

Statistical data analysis was performed with GraphPad Prism. Unpaired, two-tailed Student’s *t* tests or one-way analyses of variance (ANOVA) with Dunnett’s or Tukey’s multiple-comparison tests were used to determine statistically significant differences in growth-related phenotypes, gene expression, cell wall data, and Ca^2+^ signatures. Differences in mortality curves were assessed using log rank (Mantel-Cox) tests.
