# Supplementary figures and images for: Functional Coupling between the Unfolded Protein Response and Endoplasmic Reticulum/Golgi Ca2+-ATPases Promotes Stress Tolerance, Cell Wall Biosynthesis, and Virulence of Aspergillus fumigatus
Source: mBio. 2020 Jun 2;11(3):e01060-20. doi: 10.1128/mBio.01060-20 (PMC7267887; doi:10.1128/mBio.01060-20)

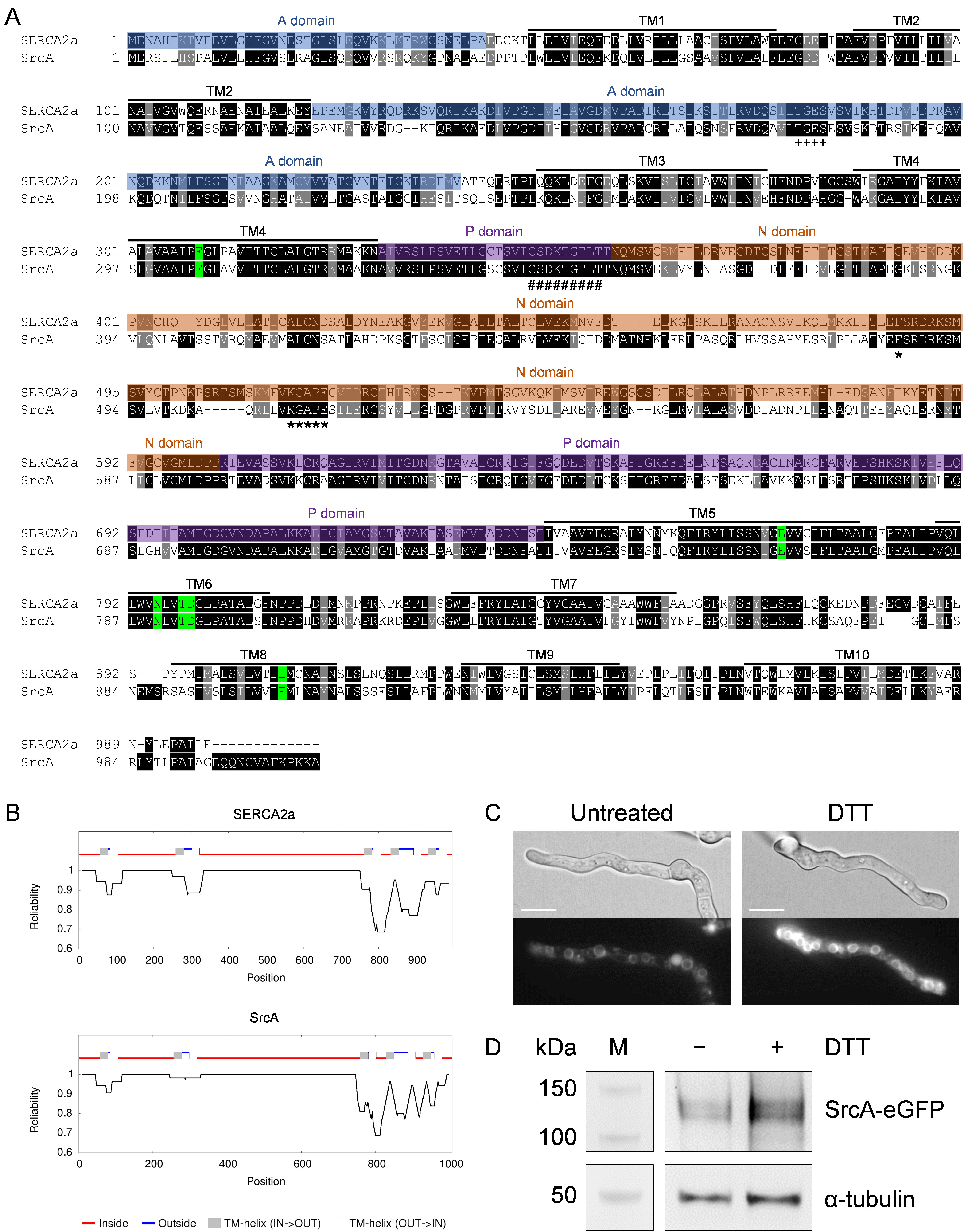

Supplement: FIG S1 [file mBio.01060-20-sf001.jpg]

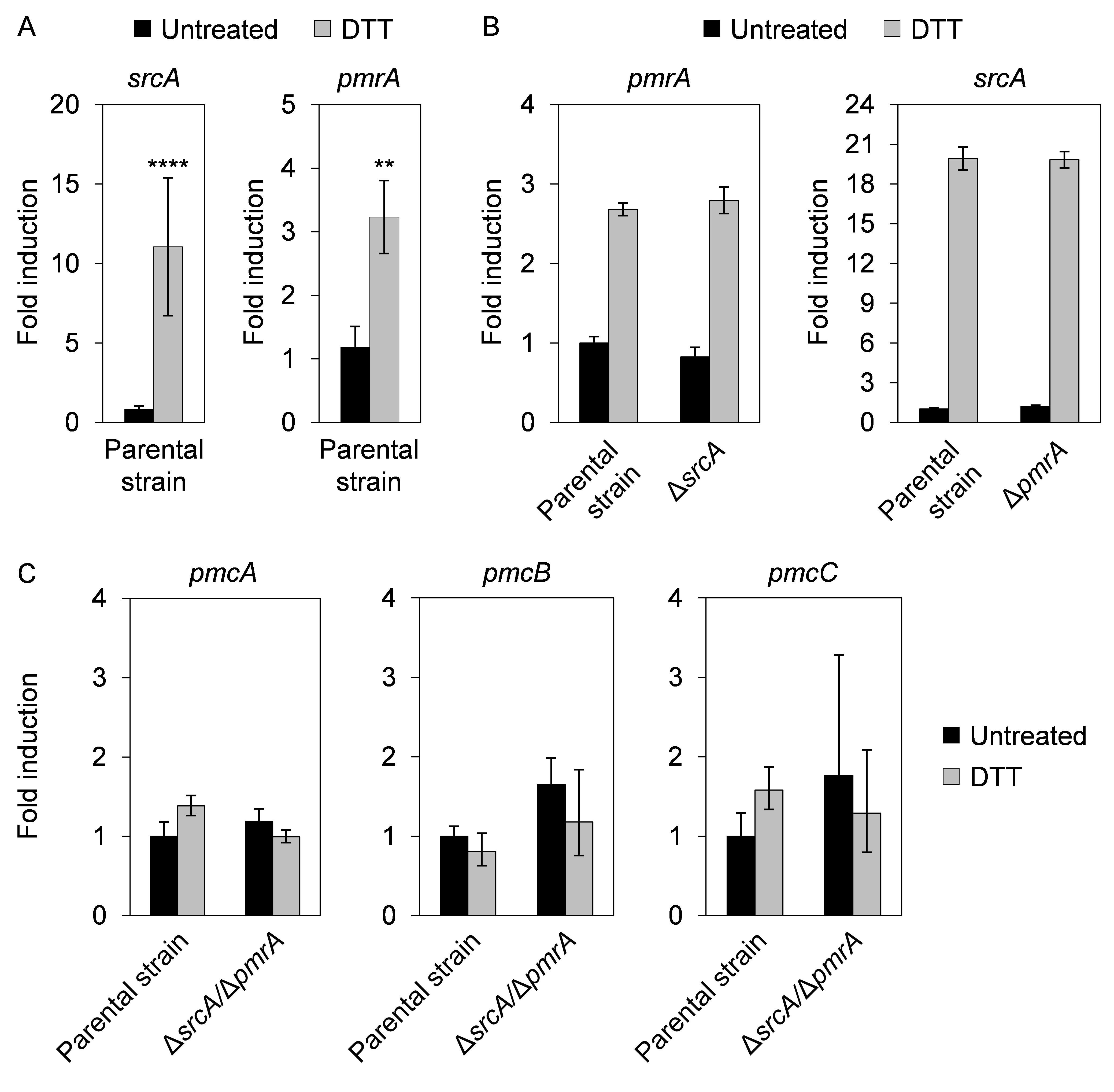

Supplement: FIG S2 [file mBio.01060-20-sf002.jpg]

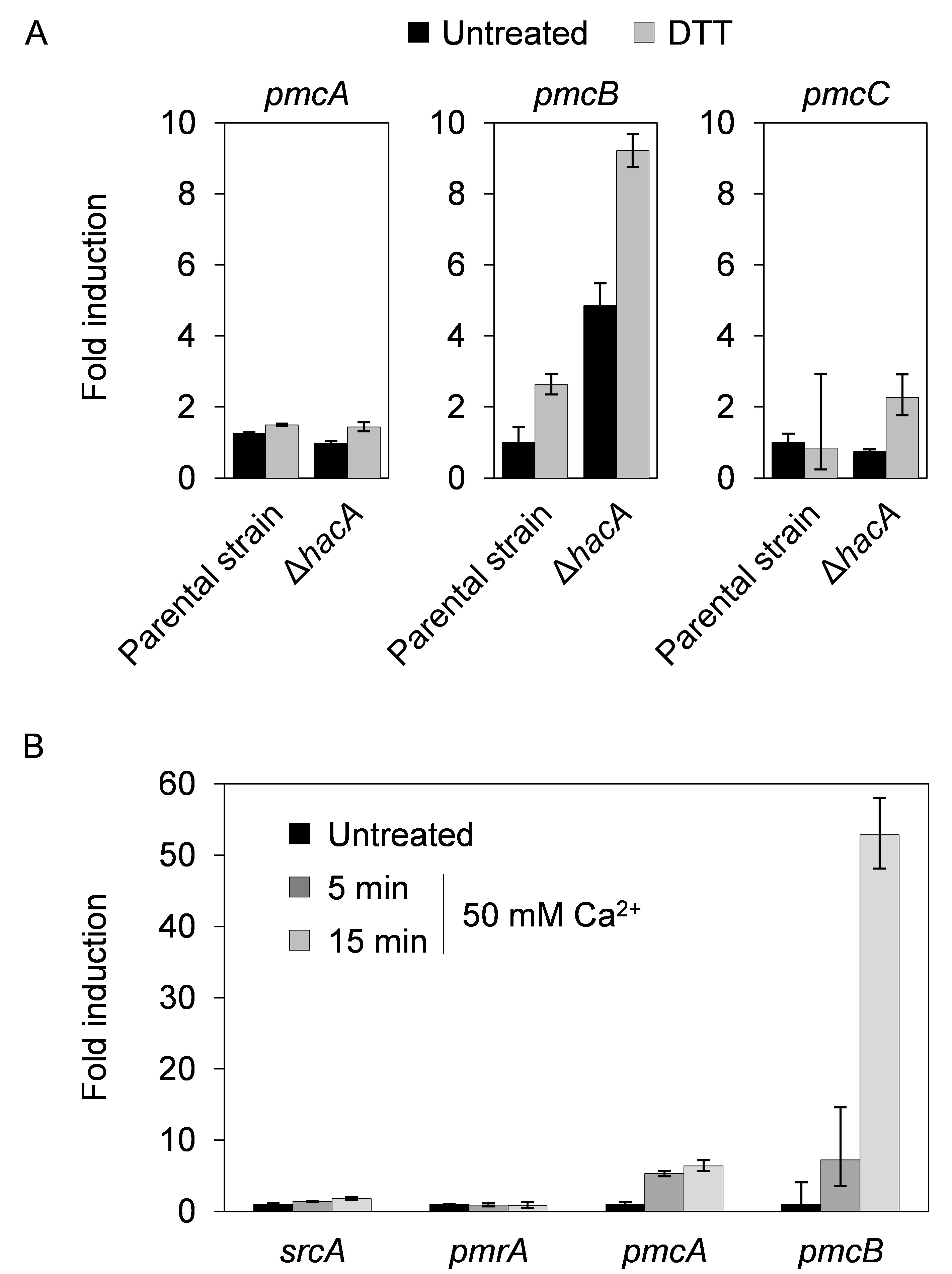

Supplement: FIG S3 [file mBio.01060-20-sf003.jpg]

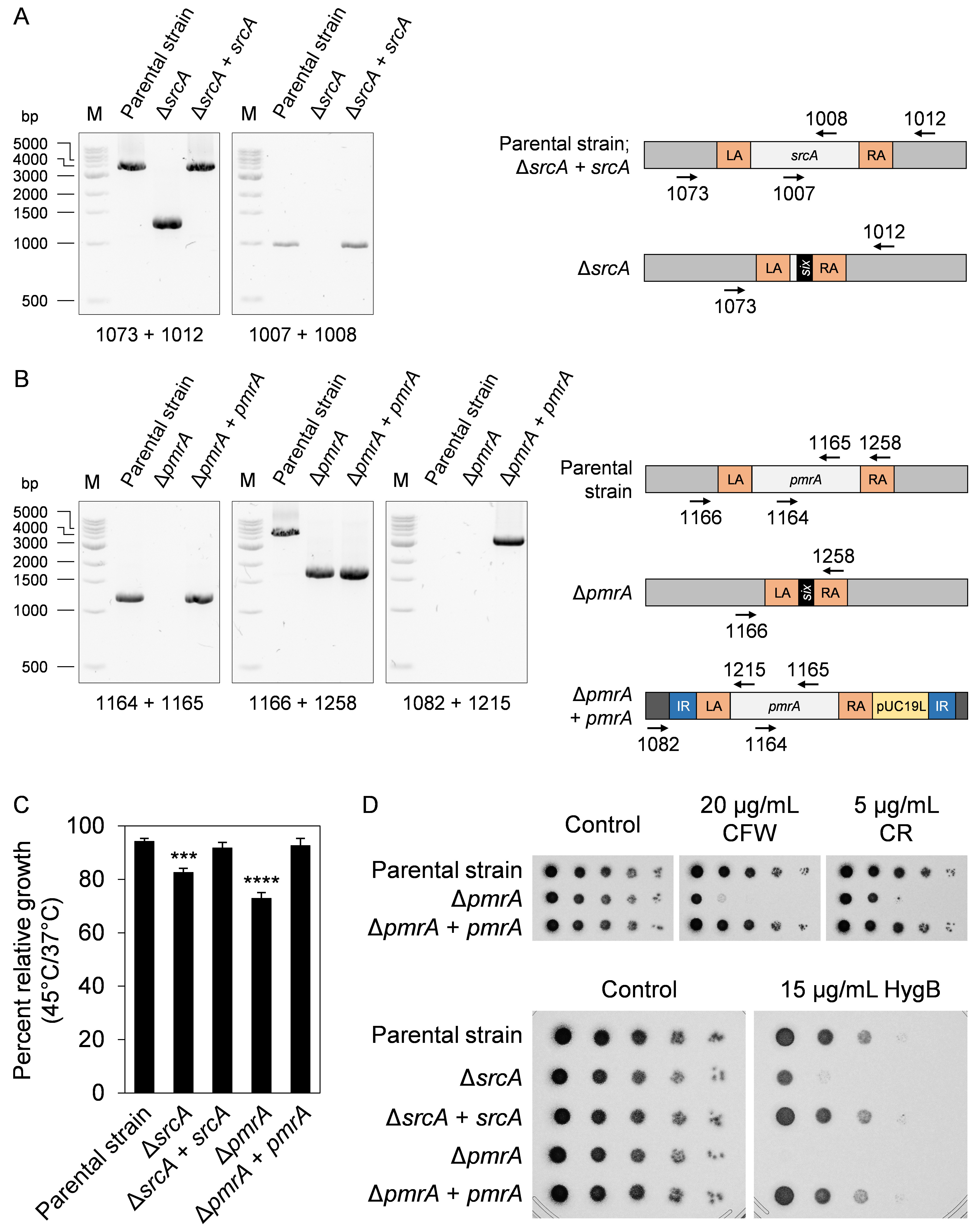

Supplement: FIG S4 [file mBio.01060-20-sf004.jpg]

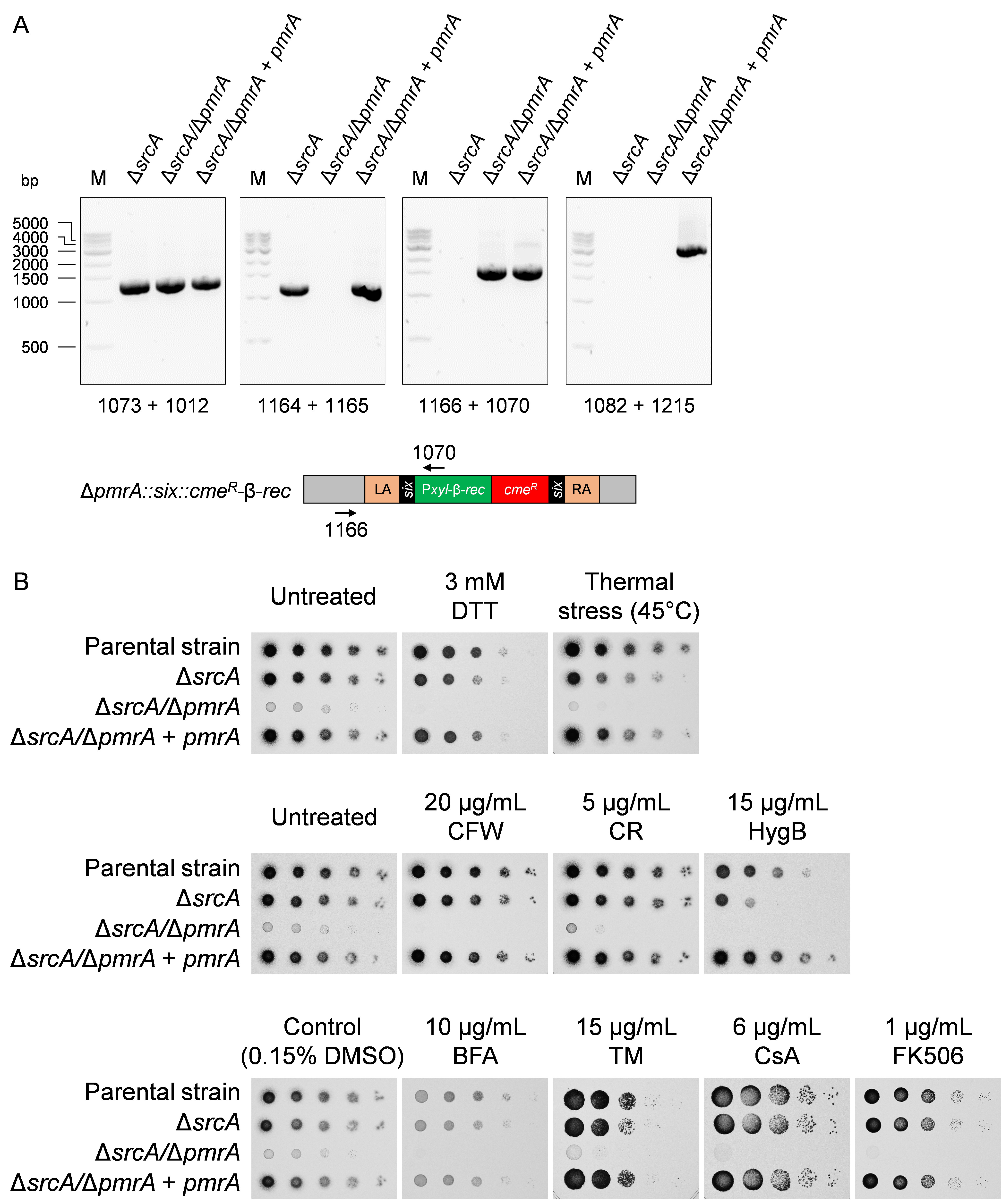

Supplement: FIG S5 [file mBio.01060-20-sf005.jpg]

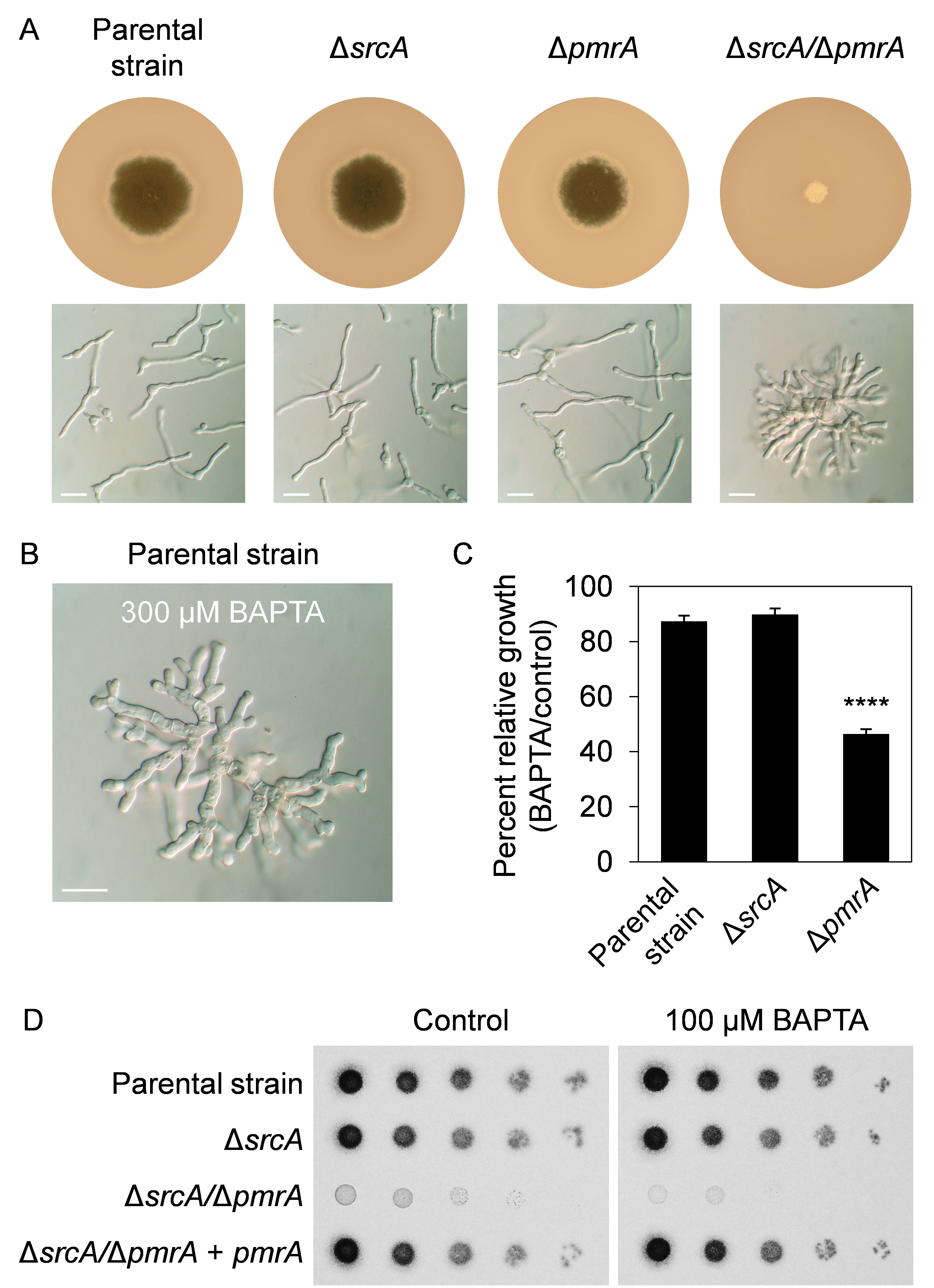

Supplement: FIG S6 [file mBio.01060-20-sf006.jpg]

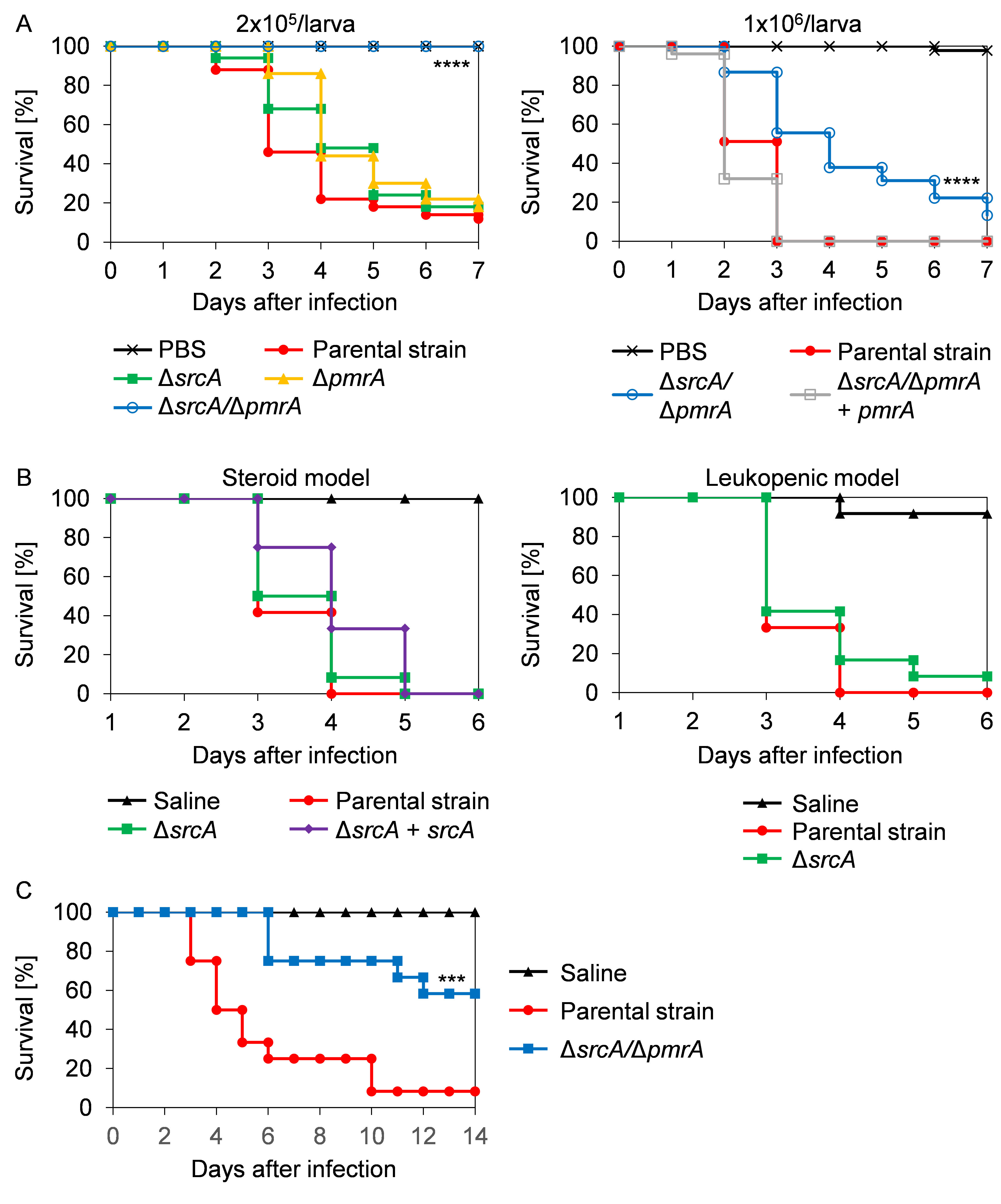

Supplement: FIG S7 [file mBio.01060-20-sf007.jpg]

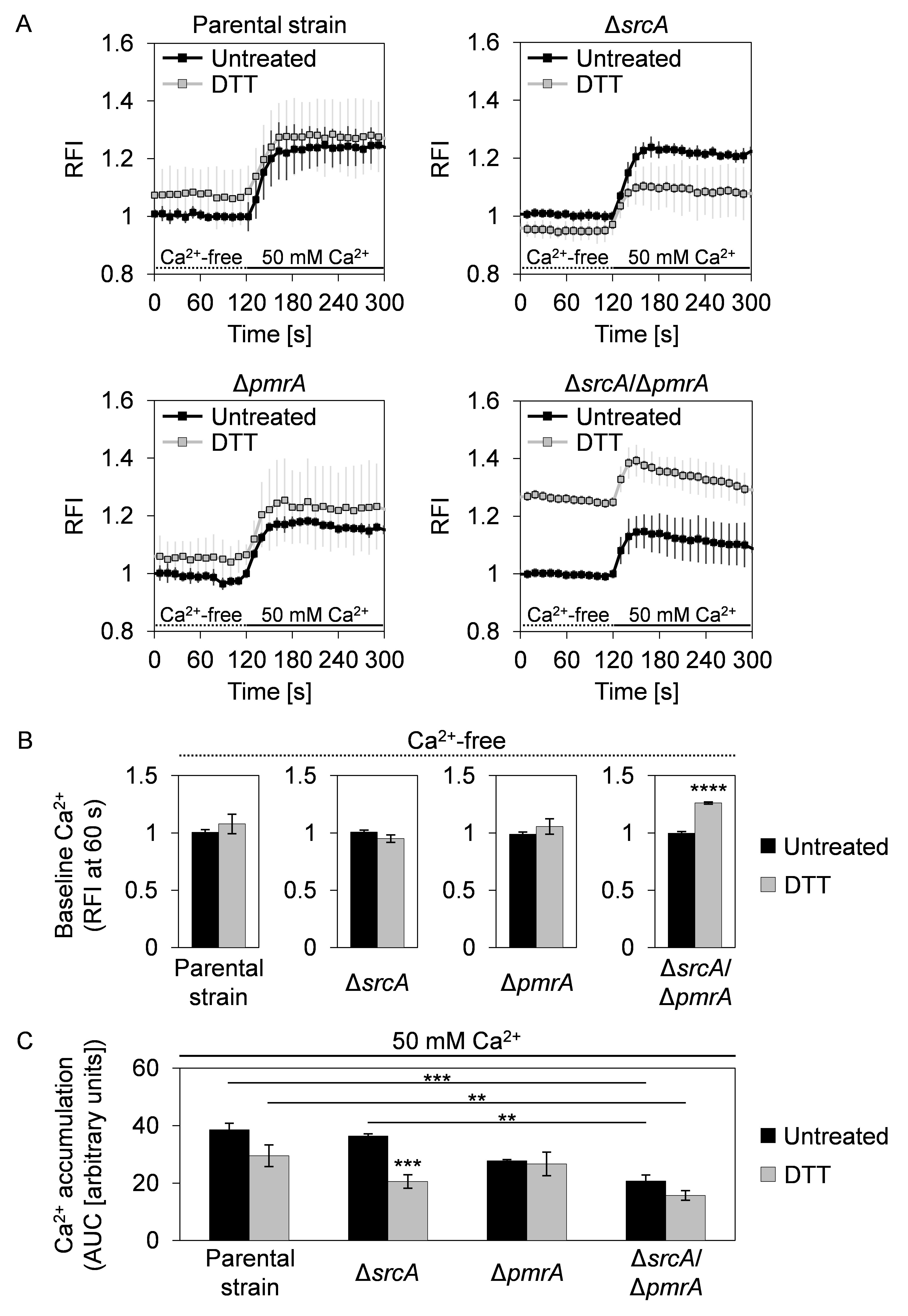

Supplement: FIG S8 [file mBio.01060-20-sf008.jpg]
