# Supplementary material for: Functional Coupling between the Unfolded Protein Response and Endoplasmic Reticulum/Golgi Ca2+-ATPases Promotes Stress Tolerance, Cell Wall Biosynthesis, and Virulence of Aspergillus fumigatus
Source: mBio. 2020 Jun 2;11(3):e01060-20. doi: 10.1128/mBio.01060-20 (PMC7267887; doi:10.1128/mBio.01060-20)
Supplement: TABLE S1 [file mBio.01060-20-st001.docx]

**TABLE S1** Strains of *Aspergillus fumigatus* used in this study.

| **Strain** | **Number** | **Genotype** | **Origin** |
| --- | --- | --- | --- |
| KU70 | 124 | ∆*akuA*::*ptrA* | Reference 60 |
| Δ*hacA* | 144 | ∆*akuA*::*ptrA*, ∆*hacA*::*hph* | Reference 14 |
| Δ*hacA* + *hacA* | 178 | ∆*akuA*::*ptrA*, ∆*hacA*::*hph*, *hacA/ble* | Reference 14 |
| Δ*hacA* | 467 | ∆*akuA*::*ptrA*, ∆*hacA*::*six* | This study |
| KU70 GCaMP5 | 750 | ∆*akuA*::*ptrA*, P*gpd1*-*gcamp5-*Tβ-*tub-six-hph-*β*-rec-six* | This study |
| Δ*hacA* GCaMP5 | 724 | ∆*akuA*::*ptrA*, ∆*hacA*::*six*, *six-*P*gpd1*-*gcamp5-*Tβ-*tub-six-hph-*β*-rec-six* | This study |
| KU80 | 399 | ∆*akuB*::*pyrG^+^* | Reference 61 |
| SrcA-eGFP | 725 | ∆*akuB*::*pyrG^+^*_,_ *srcA*::*egfp-six* | This study |
| Δ*srcA* | 402 | ∆*akuB*::*pyrG^+^*_,_ ∆*srcA*::*six* | This study |
| Δ*srcA* + *srcA* | 569 | ∆*akuB*::*pyrG^+^*_,_ ∆*srcA*::*srcA* | This study |
| Δ*pmrA* | 630 | ∆*akuB*::*pyrG^+^*_,_ ∆*pmrA*::*six* | This study |
| Δ*pmrA* + *pmrA* | 710 | ∆*akuB*::*pyrG^+^*_,_ ∆*pmrA*::*six, pmrA* | This study |
| Δ*srcA*/Δ*pmrA* | 643 | ∆*akuB*::*pyrG^+^*_,_ ∆*srcA*::*six,* ∆*pmrA*::*six-cme^R^-*β*-rec-six* | This study |
| Δ*srcA*/Δ*pmrA* + *pmrA* | 769 | ∆*akuB*::*pyrG^+^*_,_ ∆*srcA*::*six,* ∆*pmrA*::*six*-*cme^R^-*β*-rec-six, pmrA/hph* | This study |
| KU80 GCaMP5 | 585 | ∆*akuB*::*pyrG^+^*, P*gpd1*-*gcamp5-*Tβ-*tub-six* | This study |
| Δ*srcA* GCaMP5 | 480 | ∆*akuB*::*pyrG^+^*, ∆*srcA*::*six*, P*gpd1*-*gcamp5-*Tβ-*tub-six* | This study |
| Δ*pmrA* GCaMP5 | 686 | ∆*akuB*::*pyrG^+^*, ∆*pmrA*::*six*, P*gpd1*-*gcamp5-*Tβ-*tub-six* | This study |
| Δ*srcA* Δ*pmrA* GCaMP5 | 656 | ∆*akuB*::*pyrG^+^*, ∆*srcA*::*six*, ∆*pmrA*::*six-cme^R^-*β*-rec-six*, P*gpd1*-*gcamp5-*Tβ-*tub-six* | This study |
