# Supplementary material for: Functional Coupling between the Unfolded Protein Response and Endoplasmic Reticulum/Golgi Ca2+-ATPases Promotes Stress Tolerance, Cell Wall Biosynthesis, and Virulence of Aspergillus fumigatus
Source: mBio. 2020 Jun 2;11(3):e01060-20. doi: 10.1128/mBio.01060-20 (PMC7267887; doi:10.1128/mBio.01060-20)
Supplement: TABLE S2 [file mBio.01060-20-st002.docx]

**TABLE S2** List of oligonucleotides used in this study.

| **Number** | **Gene/locus/purpose** | **Nucleotide sequence (5’-3’)** |
| --- | --- | --- |
| **Molecular cloning** | | |
| 1053 | Marker module (*hph* or *cme^R^* with β-*rec*) | GCATCACTCAGGTCCTATAGGTC |
| 1054 | Marker module (*hph* or *cme^R^* with β-*rec*) | GCACTAGATGGACCATATTATGC |
| 1061 | pUC19L | GGCATGCAAGCTTGGCGTAATCATGG |
| 1062 | pUC19L | GTACCGAGCTCGAATTCACTGGCCGT |
| 1225 | *egfp* | ATGGTGAGCAAGGGCGAGG |
| 1119 | *egfp* | AGGACCTGAGTGATGCTTACTTGTACAGCTCGTCCATG |
| 1341 | *srcA-egfp* | AATTCGAGCTCGGTACCTGATTGGCCTTGTTGG |
| 1342 | *srcA-egfp* | CCTCGCCCTTGCTCACCATCGCCTTCTTGGGCTT |
| 1106 | *srcA-egfp* | TGGTCCATCTAGTGCCCTGATGTGTGGTGATGAG |
| 1107 | *srcA-egfp* | GCCAAGCTTGCATGCCCCTGGCAGATCATACCCAA |
| 1057 | ∆*srcA* | AATTCGAGCTCGGTACTGCGCACCTCATTGAACAGAAGGCAAAT |
| 1049 | ∆*srcA* | GGACCTGAGTGATGCGTCAGCTTGGATCACTCTGT |
| 1058 | ∆*srcA* | GCCAAGCTTGCATGCCTGCGCACGAACAGGCCCTTTATGTACTC |
| 1050 | ∆*srcA* | TGGTCCATCTAGTGCGGCTGATGAAGTGATGA |
| 1160 | ∆*pmrA* | AATTCGAGCTCGGTACAAATATCCAAAAAGATGCCC |
| 1161 | ∆*pmrA* | AGGACCTGAGTGATGCAAACATTCAATCAAACCGGG |
| 1162 | ∆*pmrA* | ATGGTCCATCTAGTGCTGTGCATTCGATAGAGAGGG |
| 1163 | ∆*pmrA* | GCCAAGCTTGCATGCCATGCATTTCGGGGTTGATGG |
| 1003 | ∆*srcA* complementation | CCTCATTGAACAGAAGGCAAAT |
| 1006 | ∆*srcA* complementation | CGAACAGGCCCTTTATGTACTC |
| 1311 | ∆*pmrA* complementation | aatgtattaattaaAAATATCCAAAAAGATGCCC |
| 1312 | ∆*pmrA* complementation | aatgtaggcgcgccATGCATTTCGGGGTTGATGG |
| 1077 | Intergenic region | AATTCGAGCTCGGTACTGTAGGCAGTGAAGCGATTAG |
| 1078 | Intergenic region | GATTCGAAGCCCGGGTGCACGCTACATATGCACGCA |
| 1079 | *gcamp5* construct | CCCGGGCTTCGAATCGTGGCTAC |
| 1080 | *gcamp5* construct | GCCAAGCTTGCATGCCCCCGGGCTCAAGCTTATCATCATGCAACA |
| 1047 | ∆*hacA* | GGACCTGAGTGATGCCCGGTAGACAAGATCACAGG |
| 1048 | ∆*hacA* | TGGTCCATCTAGTGCATTGCAGCTGGCTGTTAGTG |
| 1055 | ∆*hacA* | AATTCGAGCTCGGTACTGCGCACCTTCGCTACAGACACATGG |
| 1056 | ∆*hacA* | GCCAAGCTTGCATGCCTGCGCACCTCTATCGCACTACTAGCG |
| **Confirmatory PCR analysis** | | |
| 1007 | *srcA* | GGCTAAGGGTGCCATTTACTA |
| 1008 | *srcA* | GTGATAACAATCACGCGAATACC |
| 1012 | *srcA* | GCACAGGCCATCGTCTATATC |
| 1070 | Marker module | GGACTAAGGCTTCTGGCTAATG |
| 1073 | *srcA* | GGCATCATCCTACTGTCTAAATCC |
| 1082 | Intergenic region | GTCATTTCGAGTCGCGTTATTG |
| 1139 | Intergenic region | CAACCTGCATGGCTCTTAATC |
| 1183 | Intergenic region | GCAGAGATTCGGCACCGGCTAGTGG |
| 1164 | *pmrA* | GGATATCGAACTGGAGGATCTTGCG |
| 1165 | *pmrA* | TTGGTTAGGTAAGTATCACACCGGG |
| 1166 | *pmrA* | CAGCTGACCACTAAGTCGTAGAGCC |
| 1215 | *pmrA* | AGTTCGTTTGGACCATCTCG |
| 1258 | *pmrA* | TCCGAATCAAAGGATCAGCATGG |
| 1114 | *srcA-egfp* | GCTTACATCCGTCCTCACTAAG |
| 1115 | *srcA-egfp* | GTACACCTTCACTTCCTCATCC |
| 1081 | *gcamp5* construct | CCCAGCTCCTTGGTTGTTAT |
| 1225 | *egfp; gcamp5* | ATGGTGAGCAAGGGCGAGG |
| 1287 | pUC19L | CGCCAGGGTTTTCCCAGTCACGAC |
| **Gene expression analysis (RT-qPCR)** | | |
| 1252 | *18S rRNA* (F) | ACTGATACGGGGCTCTTTTG |
| 1253 | *18S rRNA* (R) | GACTTGCCCTCCAATTGTTC |
| 1232 | *srcA* (F) | CGGTGCTGCTACTGTTTTTG |
| 1233 | *srcA* (R) | ACTTGTGGAAATGGGACAGC |
| 1212 | *pmrA* (F) | AACCATTCGAGGTCCACAAC |
| 1213 | *pmrA* (R) | GCCGGAGAATTTGCACTAAC |
| 1380 | *pmcA* (F) | TTGGGTATGGGCTCGATTAG |
| 1381 | *pmcA* (R) | CACTGCCATGCATTTACGTC |
| 1382 | *pmcB* (F) | AAACAATCTCGTCCGTGTCC |
| 1383 | *pmcB* (R) | CAGCGACGACAGTCATTTTG |
| 1384 | *pmcC* (F) | TGACCATGTGGAAGCTGATG |
| 1385 | *pmcC* (R) | TTGAAGATGACGGTGTCGAG |
| 1355 | *hacA^u/i^* (F) | TTGTTCAAGCAAGAAGGTGATG |
| 1356 | *hacA^u^* (R) | GTCGCACAACACCGCTG |
| 1354 | *hacA^i^* (R) | ACTGACACTGCAGGATGTTGTG |
| 1218 | *bipA* (F) | TGATGAAGAGCGTCTGGTTG |
| 1219 | *bipA* (R) | TCTGGACATCCTTGTCATCG |
| 1271 | *pdiA* (F) | TCAAGGTCGATTGCACTGAG |
| 1272 | *pdiA* (R) | GGAGGCAAAGTAACCGATGA |
